# Comparison of the therapeutic effects of traditional Chinese medicine exercise therapies on blood pressure, lipids, and sleep quality among older patients suffering from hypertension: a systematic review and network meta-analysis

**DOI:** 10.3389/fcvm.2026.1707525

**Published:** 2026-03-11

**Authors:** Binbin Zhang, Mingyue Jiao, Xiaohui Zhao, Fangfang Wen, Jifeng Long, Jian Li, Mohd Taib Harun, Xianzhi Xie

**Affiliations:** 1 Faculty of Sports and Leisure, Guangdong Ocean University, Zhanjiang, Guangdong, China; 2 Faculty of Education and Liberal Arts, INTI International University, Nilai Negeri Sembilan, Malaysia; 3 School of Teacher Education, Hezhou University, Hezhou, Guangxi, China; 4 Department of Physical Education, Shanxi University of Science and Technology, Jincheng, Shanxi, China

**Keywords:** hypertension, network meta-analysis, older patients, therapies, traditional Chinese medicine exercise

## Abstract

**Purpose:**

This study aimed to compare the therapeutic effects of traditional Chinese medicine exercise therapies on blood pressure, blood lipid, quality of life, Pittsburgh sleep quality index (PSQI), and heart rate (HR) among older patients suffering from hypertension through a network meta-analysis (NMA).

**Methods:**

A total of eight databases (in Chinese or English)—PubMed, Embase, Cochrane Library, Web of Science, CNKI, Wanfang Data, CQVIP, and China Biology Medicine disc (CBM disc)—were systematically retrieved up to 7 August 2025, in order to determine eligible randomized controlled trials (RCTs) evaluating the therapeutic effects of different traditional Chinese medicine exercises on blood pressure in older populations. Literature screening was performed based on inclusion and exclusion criteria. Data extraction and quality evaluation were performed through Stata 17.0 and R (version 4.3.3) within this NMA framework.

**Results:**

This study included 44 RCTs, involving 3,478 older patients suffering from hypertension. Wuqinxi (WQX) and Qigong (QG) showed equivalent effects in reducing systolic blood pressure (SBP) and diastolic blood pressure (DBP). Baduanjin (BDJ) was most effective in improving total cholesterol, low-density lipoprotein cholesterol (LDL-C), and PSQI. Tai Chi demonstrated the most significant effects in improving triglyceride (TG). The surface under the cumulative ranking curve ranking indicated that WQX (85.7%) and QG (76.8%) had the highest potential to be the best exercise interventions for improving SBP and DBP. BDJ was the most beneficial intervention for improving TC (95.1%), LDL-C (95.1%), and PSQI (80.5%).

**Conclusion:**

Traditional Chinese medicine exercises exhibit different therapeutic effects in older patients with hypertension. Patients engage in targeted exercises based on their own conditions. WQX, QG, and BDJ may be the most effective therapies, while TC (Tai Chi) is the least effective intervention.

**Systematic Review Registration:**

https://www.crd.york.ac.uk/PROSPERO/view/CRD420251049049, Registration No: CRD420251049049.

## 1 Introduction

Hypertension is a major global health concern and the primary contributor to worldwide mortality and morbidity, particularly in China. According to the *Annual Report on Cardiovascular Health and Diseases in China (2023)* (1), the prevalence of hypertension exceeds 50% among individuals aged 60 years and above, and reaches 90% in those over 80 years. It is projected that hypertension cases in China will rise to 400 million by 2030 (2). Hypertension stands as a major risk factor for cardiovascular diseases and is closely linked to various metabolic diseases, including diabetes mellitus and obesity (3, 4). Research has indicated that approximately 30% of patients with hypertension also suffer from diabetes mellitus or metabolic syndrome, placing them at a 2-fold increased risk of heart disease compared to healthy adults (5). Furthermore, long-term hypertension is strongly linked to adverse health outcomes such as stroke and cardiac abnormalities, severely impairing mental health and social functioning in older patients and thereby lowering their quality of life (6). Consequently, exploring more comprehensive and effective interventions for older adults with hypertension—particularly approaches integrating traditional and modern medicine—has become a crucial topic in current research regarding public health.

Existing interventions for hypertension primarily focus on medication, aerobic exercise, and resistance training; however, these approaches have certain limitations. Long-term use of antihypertensive medications may cause adverse effects, comprising electrolyte imbalance and renal impairment, leading to poor compliance from patients (7). Older adults often suffer from decreased muscle strength, joint degeneration, reduced bone density, and weakened cardiovascular function. In this context, high-intensity exercise without proper assessment and individualized design may elevate the risk of joint injury, fractures, falls, and cardiovascular events (8). Research has shown that among older adults engaging in moderate- to high-intensity resistance exercise, a positive correlation is observed between exercise intensity and both systolic blood pressure (SBP) and brachial-ankle pulse wave velocity (baPWV) (9). During high-intensity exercise, sympathetic nerve activity increases significantly. In older patients with hypertension, such abrupt fluctuations in blood pressure may trigger acute cardiovascular events, including myocardial infarction and stroke (10, 11).

Traditional Chinese medicine exercise therapies—comprising Tai Chi, Baduanjin, Qigong, and Wuqinxi—integrate body adjustment, breath control, and mind regulation, embodying a holistic approach that harmonizes body, breath, and mind. These exercises are suitable for older adults as they can be practiced over the long term (12). Recent studies have consistently demonstrated that exercise assists in lowering blood pressure through mechanisms such as improved endothelial function, regulation of autonomic nervous balance, and reduction of inflammatory markers (13, 14). Research has found that a 3-month intervention using Baduanjin reduced SBP by an average of 4.36 mmHg in older patients with hypertension (13), while Tai Chi showed significant effects in improving diastolic blood pressure (DBP) (15). Both exercises also contributed to enhanced quality of life, heart rate stability, and autonomic nervous function (16). For older patients with hypertension, traditional Chinese exercises, compared with high-intensity aerobic exercise or resistance training, are characterized by lower intensity, gentler movements, absence of acute hemodynamic stress, and better adherence. Therefore, the cardiovascular risk is reduced and the quality of life is improved among older patients.

However, current evidence largely focuses on comparing single exercises against conventional therapy (such as Tai Chi vs. no exercise) or focuses on individual blood pressure metrics, providing limited direct and indirect comparisons across different traditional Chinese medicine exercise therapies. Consequently, the relative effectiveness of these therapies remains uncertain—particularly among older adults, who commonly have multiple comorbidities, may be more susceptible to intervention-related adverse effects, and often prioritize patient-centered outcomes. Moreover, prior syntheses rarely provide a comprehensive assessment of outcomes central to geriatric care, such as lipid profiles, sleep quality, and health-related quality of life, which are increasingly emphasized in contemporary hypertension management and long-term risk reduction. To address these gaps, we conducted a systematic review and network meta-analysis (NMA) of elderly patients with hypertension (≥60 years), integrating evidence from 44 randomized controlled trials. We assessed clinically meaningful outcomes—including systolic and diastolic blood pressure, lipid parameters, Pittsburgh sleep quality index (PSQI) scores, quality-of-life measures, and heart rate—and used the surface under the cumulative ranking curve (SUCRA) to rank TCM exercise therapies and provide an interpretable hierarchy of relative preference. By evaluating which exercise is most effective, this study aims to inform evidence-based, personalized exercise prescriptions in clinical practice and improve health outcomes in older adults with hypertension.

## 2 Methods and materials

This systematic review followed the Preferred Reporting Items for Systematic reviews and Meta-Analyses (PRISMA) guidelines (17) and was registered in the International Prospective Register of Systematic Reviews (PROSPERO) in May 2025 (Registration No: CRD420251049049).

### 2.1 Literature retrieval

A systematic retrieval of relevant literature was performed across eight databases: PubMed, Embase, Cochrane Library, Web of Science, CNKI, Wanfang Data, CQVIP, and China Biology Medicine disc (CBM disc). Search strategies combined subject terms and free terms as follows: hypertension (“high blood pressure,” “HTN,” “hypertension,” “hypertensive disease,” “hypertensive effect,” “hypertensive reaction,” and “hypertensive response”), traditional Chinese medicine exercise (“changing tendon exercise,” “five mimic animal exercise,” “hexagram boxing,” “shadow boxing,” “Shadowboxing,” “Six Words,” “T ai Chi,” “Tai Chi,” “Tai Ji,” “Taiji,” “taijiquan,” “Yi jin jing,” “Yi jinjing,” and “Yijin Jing”), and randomized controlled trial (“Randomized controlled trial” and “RCT”). The retrieval period was restricted to the time from the inception of the databases to 7 August 2025. We conducted three supplementary snowballing searches: (a) screening the reference lists of included studies; (b) identifying articles that cited the included studies (e.g., via Google Scholar); and (c) locating related studies using the “Similar articles/Find similar” features in PubMed and Embase. Before implementation, the search strategies were peer reviewed by independent members of the author team in accordance with the PRESS 2015 checklist. Full search strategies are provided in Supplementary Table S1.

### 2.2 Inclusion and exclusion criteria

#### 2.2.1 Inclusion criteria

1. Study subject: The subjects were older patients (≥60 years) with hypertension diagnosed according to World Health Organization (WHO) or Chinese hypertension guidelines, with hypertension primarily defined by office BP (SBP ≥140 mmHg and/or DBP ≥90 mmHg). Common comorbidities, such as obesity and insomnia, were permitted.
2. Intervention: The intervention group practiced a single type of traditional Chinese medicine exercise—including Tai Chi (TC), Baduanjin (BDJ), Qigong (QG), and Wuqinxi (WQX) (Table 1)—for at least 2 weeks. The control group underwent conventional antihypertensive medications, conventional antihypertensive medications combined with health education, or conventional antihypertensive medications combined with regular walking.
3. Outcome: Primary outcomes included SBP and DBP. Secondary outcomes comprised quality of life (QOL), PSQI, total cholesterol, TG, HDL-C, LDL-C, and heart rate (HR).
4. Study type: The study type was RCT.

#### 2.2.2 Exclusion criteria

1. Duplicate publications, review articles, letters to the editor, conference abstracts, studies reporting acute effects of one single exercise session, or animal studies were excluded.
2. Studies combining exercise interventions with other interventions, such as diet, were also excluded.

### 2.3 Literature screening and data extraction

Literature screening was performed using EndNote X9. Two researchers independently screened the literature based on the inclusion and exclusion criteria. If the same trial was covered by multiple publications, the most comprehensive report was selected as the main reference.

**Table 1:** Characteristics of the exercises included in the network meta-analysis.

Type	Definition
C (the control group)	The control group receives traditional antihypertensive medications, traditional antihypertensive medications combined with health education, or traditional antihypertensive medications combined with regular walking.
TC (Tai Chi)	Tai Chi refers to a martial art based on the principles of Yin–Yang philosophy, traditional Chinese meridian theory, and qigong. It emphasizes using softness to overcome hardness and relies on intention rather than force, including 24 movements: (1) beginning from, (2) parting the wild horse's man to left and right, (3) white crane spreads its wings, (4) brush knee and twist step-left (right) style, (5) hand strums the lute, (6) step back and whirl arms on both sides, (7) grasp the birds tail (left style), (8) grasp the birds tail (right style), (9) single whip, (10) cloud hand swing, (11) single whip, (12) high pat on horse, (13) kick with right heel leading, (14) strike opponent's ears with both fists, (15) turn body and kick with left heel leading, (16) push down and stand on one leg (left style), (17) push down and stand on one leg (right style), (18) fair lady works at the shuttle, (19) needle at the bottom of the sea, (20) flash the arm, (21) turn, deflect downward, parry and bunch, (22) apparent close-up, (23) cross hands, and (24) closing form.
BDJ (Baduanjin)	Baduanjin is composed of eight independent and well-arranged movements (segments): (1) holding up the sky with both hands to regulate the sanjiao (triple burner), (2) drawing the bow to shoot the hawk, (3) lifting the arms to regulate the spleen and stomach, (4) looking backwards to relieve the five fatigues and seven injuries, (5) punching with a fierce gaze, (6) holding the feet with two hands to strengthen the kidney and lumbar area, (7) clearing the heart fire by shaking the head and wagging the tail, and (8) standing on the tiptoes eliminates illnesses.
QG (Qigong)	Qigong refers to a comprehensive physical and mental exercise system that cultivates, enhances, guides, and utilizes “qi” (life energy) through the integration of body adjustment, breath control, and mind regulation. Qigong includes dynamic exercises and static exercises.
WQX (Wuqinxi)	Wuqinxi is a set of biomimetic exercise routines created by imitating the expressions, movements, and habits of five animals: ape, bear, bird, deer, and tiger.

Data from the included literature were independently extracted by two researchers using a standardized, prespecified, and piloted sheet. Based on the inclusion and exclusion criteria, data were extracted concerning participants, characteristics, interventions, comparisons, and outcomes.

### 2.4 Risk of bias and GRADE assessment

The revised Cochrane Risk of Bias 2 (RoB 2) tool for randomized trials was used to evaluate the risk of bias in the included studies (18). The RoB2 tool evaluated bias from five aspects, comprising (i) the randomization process, (ii) the established intervention, (iii) the missing data of outcomes, (iv) the measurement of outcomes, and (v) the selective reporting of outcomes. The risk of bias for each aspect was judged as low risk, some concerns, or high risk. Two independent and blinded researchers evaluated the risk of bias among all included studies, with any disputes settled through discussion with a third independent researcher.

The quality of the evidence for the outcome indicators was graded using the GRADE assessment system (https://gradepro.org/) (19). The evidence was upgraded or downgraded according to the factors affecting its quality, comprising risk of bias, inconsistency, indirectness, precision, and publication bias.

### 2.5 Statistical analysis

A frequentist NMA was conducted using STATA 17.0 (StataCorp, College Station, TX, USA) (20). Since different scales and units were used to measure QOL among the included studies, standardized mean difference (SMD) was adopted as the effect indicator for QOL and PSQI, and mean difference (MD) served as the effect indicator for SBP, DBP, total cholesterol, TG, HDL-C, LDL-C, and HR. The I^2^ was used to assess the level of heterogeneity, with an *I*^2^ value of >50% or a *p* value of ≤0.10 indicating significant heterogeneity (21).

The relationships among exercise interventions were illustrated by network plots, where lines connecting nodes stood for direct head-to-head comparisons among interventions. The size of each node and the thickness of each line connecting the nodes were positively correlated with the number of included studies. A network contribution graph was generated to compute the contribution of each direct comparison.

Transitivity was evaluated by examining the inclusion criteria for individual studies, by determining whether all participants in the network could hypothetically be randomized to any of the interventions, and by using consistency models (17). Transitivity is a key assumption of NMA, referring to the belief that indirect comparisons provide valid estimates of unobserved direct comparisons (22), and that all studies have effect modifiers which are evenly distributed (23). The inconsistency factors (IFs) with a 95% confidence interval (CI) were calculated to assess the consistency within each closed loop, with consistency manifested by a 95% CI lower limit equal to 0 (24). The inconsistency model was employed to examine global inconsistency. When global inconsistency was not significant (*p* > 0.05), a consistency model was adopted (25). Node-splitting analysis was leveraged to check for local inconsistency, and the results were deemed reliable (*p* > 0.05).

The SUCRA was leveraged to rank and compare the therapeutic effects of the different exercises (26). SUCRA values ranged from 0 to 100, and a higher SUCRA value indicated a more effective intervention (27). Publication bias was evaluated using comparison-correction funnel plots and Egger's test. To assess the robustness of the findings, we conducted sensitivity analysis based on the overall RoB 2 assessment, restricting inclusion to studies judged to have a low overall risk of bias.

## 3 Results

### 3.1 Study screening and characteristics of the included studies

**Figure 1:**
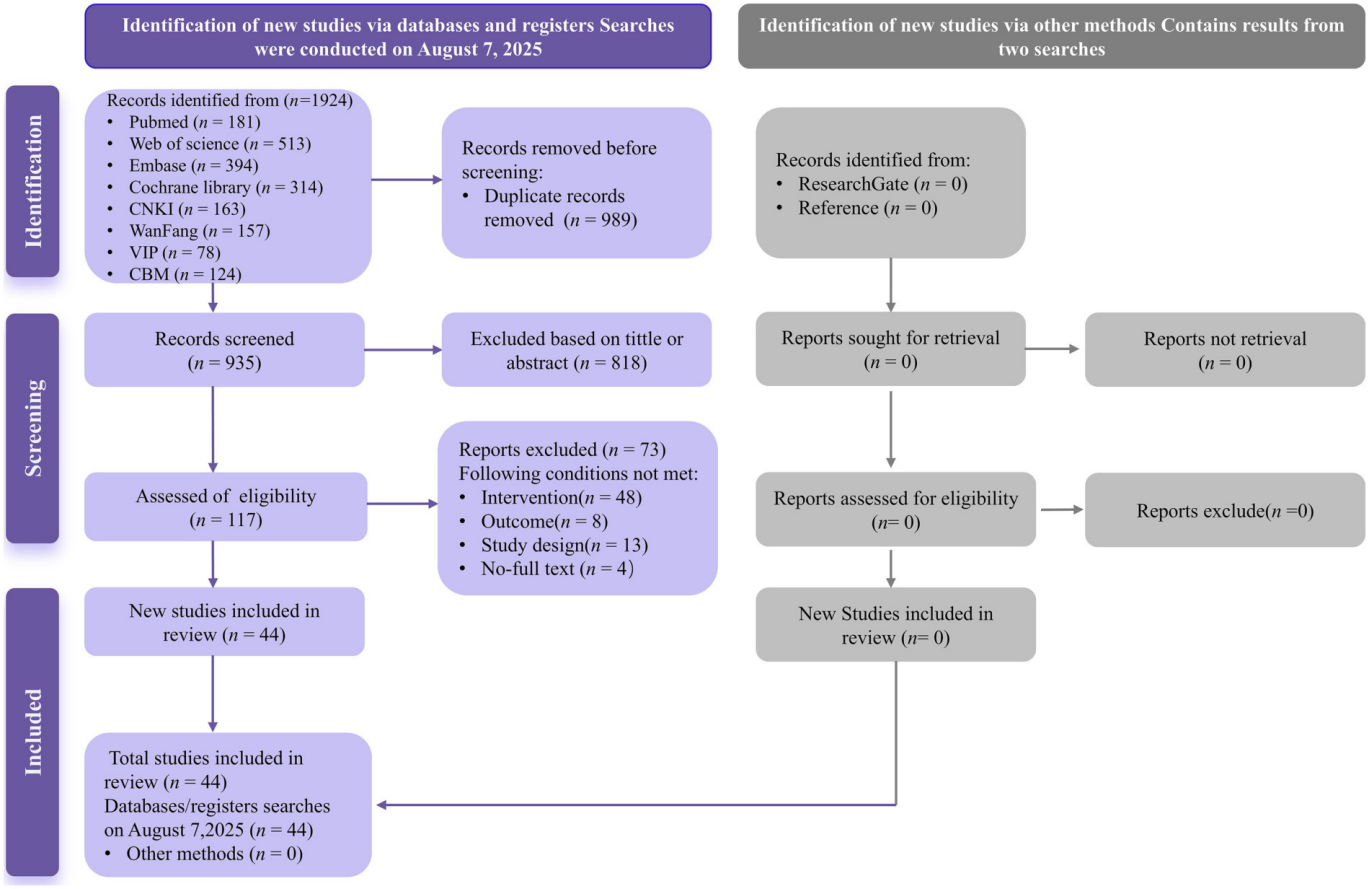
PRISMA flow diagram depicting the study selection.

The literature screening process is illustrated in Figure 1. A total of 1,924 studies that might meet the inclusion criteria were retrieved. After the elimination of 989 duplicates, the removal of 818 studies based on titles and abstracts, and the exclusion of 73 studies through reviewing the full texts, eventually, 44 studies were ultimately included in this study.

Table 2 presents the characteristics of the included studies. These studies were all published between 2009 and 2024, predominantly in China. In total, 3,478 older patients with hypertension were included in the analysis: 1,808 in the intervention group (C + TC, *n* = 823; C + BDJ, *n* = 756; C + QG, *n* = 70; C + WQX, *n* = 159) and 1,670 in the control group. Approximately 56.3% (1,959) of the participants were men. Two studies only involved women, two only involved men, 34 studies included both men and women, and the remaining six studies failed to report the sex ratio. The average duration of the traditional Chinese medicine exercise therapies was 17 weeks, ranging from 2 to 26 weeks, and over half (90.9%) of the studies indicated that the exercise intervention lasted 12 weeks.

**Table 2:** Characteristics of the included studies.

Author	Year	Province	Intervention	*N* (M/F)	Age: mean ± SD or range	Course of disease: mean ± SD or range	Course of treatment	Outcomes
Wang et al. (36)	2019	Fujian	C + TC	50 (20/30)	67.6 ± 4.5	4.2 ± 1.9	3mos	SBP, DBP, TG, TC, LDL-C, HDL-C
			C	50 (22/28)	67.4 ± 4.2	4.2 ± 1.7		
Liu et al. (37)	2018	Heilongjiang	C + TC	35（18/17）	62.4 ± 2.4	3.3 ± 0.3	6mos	SBP, DBP, SF-36
			C	35（19/16）	63.1 ± 2.1	3.4 ± 0.5		
Chen (38)	2016	Beijing	C + QG	30 (−)	66.3 ± 5.8	-	6mos	SBP, DBP, TG, TC, LDL-C, HDL-C
			C	30 (−)	66.3 ± 5.8	-		
Xiao et al. (39)	2016	Beijing	C + BDJ	24 (−)	65.6 ± 7.8	-	6mos	SBP, DBP, TG, TC, LDL-C, HDL-C
			C	24 (−)	65.6 ± 7.8	-		
Feng et al. (40)	2018	Sichuan	C + TC	36 (19/17)	66.33 ± 4.74	-	12W	SBP, DBP, TG, TC, LDL-C, HDL-C
			C	37 (14/23)	67.51 ± 4.09	-		
Ma et al. (41)	2018	Guangdong	C + TC	55 (38/17)	69 ± 9.37	-	6mos	SBP, DBP, SF-36
			C	58 (40/18)	69 ± 9.37	-		
Hedo (40)	2021	California	C + TC	102 (26/76)	72.16 ± 7.93	-	12W	SBP
			C	85 (26/59)	73.21 ± 7.80	-		
Zhang (42)	2017	Sichuan	C + TC	36 (19/17)	66.33 ± 4.74	10.38 ± 8.50	12W	SBP, DBP, TG, TC, LDL-C, HDL-C Du's Hypertension QOL Scale
			C	37 (14/23)	67.51 ± 4.09	10.50 ± 7.93		
Pan et al. (43)	2010	Guangdong	C + BDJ	24 (14/10)	62.1 ± 5.8	1.5 + 1.2	24W	SBP, DBP, TG, TC, HDL-C
			C	24 (13/11)	61.4 ± 7.1	1.7 + 0.8		
Lin et al. (44)	2014	Fujian	C + BDJ	27 (14/13)	61.26 ± 3.74	1.16 ± 0.39	12W	SBP, DBP
			C	28 (12/16)	62.03 ± 3.51	1.60 ± 0.36		
Liang et al. (45)	2016	Guangdong	C + BDJ	30 (17/13)	68.1 ± 10.1	9.2 ± 2.6	3mos	SBP, DBP, simple self-designed QOL scale
			C	30 (16/14)	70.5 ± 10.2	11.9 ± 5.8		
Luo (46)	2021	Sichuan	C + BDJ	40 (24/16)	66.54 ± 10.32	8.72 ± 2.77	12W	SBP, DBP
			C	40(27/13)	67.32 ± 9.46	8.68 ± 2.92		
Jiang (47)	2019	Zhejiang	C + BDJ	50 (25/25)	64.67 ± 3.15	-	12W	SBP, DBP
			C	50 (26/24)	65.23 ± 3.23	-		
Zheng et al. (48)	2021	Fujian	C + BDJ	38 (23/15)	72.5(68-76.25)	14.26 ± 7.39	12W	SBP, DBP, SF-12
			C	38 (17/21)	71(67.75-75)	11.74 ± 7.33		
Zheng et al. (49)	2021	Fujian	C + BDJ	34 (15/19)	70.88 ± 5.56	11.31 ± 2.96	12W	PSQI, SBP, DBP, HR
			C	34 (10/24)	70.94 ± 6.34	11.08 ± 2.99		
Yang et al. (50)	2021	Fujian	C + BDJ	33 (10/23)	63.64 ± 2.47	4.15 ± 1.75	12W	SBP, DBP
			C	34 (8/26)	62.76 ± 1.92	3.99 ± 1.63		
Tan et al. (51)	2022	Zhejiang	C + BDJ	42 (22/20)	68.93 ± 7.21	11.73 ± 3.20	12W	SBP, DBP, PSQI
			C	42 (19/23)	68.95 ± 7.21	11.59 ± 3.16		
Fan et al.(52)	2021	Fujian	C + BDJ	38 (21/17)	71.87 ± 0.76	8.32 ± 2.57	12W	SBP, DBP, SF-12
			C	38 (19/19)	71.95 ± 0.97	8.04 ± 2.88		
Zheng et al. (53)	2014	Fujian	C + BDJ	27 (13/14)	69.23 ± 3.72	9.13 ± 3.69	12W	SBP, DBP
			C	28 (16/12)	70.06 ± 3.51	8.30 ± 4.36		
Chen (54)	2016	Shanghai	C + BDJ	28 (15/13)	69.98 ± 3.11	8.123 ± 3.53	12W	SBP, DBP
			C	28 (14/14)	70.29 ± 1.77	8.612 ± 3.32		
Tang (55)	2009	Hunan	C + TC	16 (10/6)	63.65 ± 8.71	-	6mos	SBP, DBP
			C + QG	16 (12/4)	63.84 ± 8.12	-		
			C	16 (9/7)	62.79 ± 7.4	-		
Yi et al. (56)	2015	Liaoning	C + TC	25 (25/0)	67. 07 ± 4. 45	-	20W	TG, TC, LDL-C, HDL-C
			C	15 (15/0)	69. 79 ± 6. 66	-		
Zhang et al. (57)	2024	Beijing	C + BDJ	48 (−)	60-80	-	24W	SBP, DBP
			C	47 (−)	60-80	-		
Xu (58)	2015	Anhui	C + TC	50 (25/25)	69.38 ± 7.41	-	8W	SBP, DBP
			C	50 (26/24)	69.54 ± 7.37	-		
Hu et al. (59)	2015	Shanxi	C + TC	55 (25/30)	64.10 ± 7.03	5.12 ± 2.45	3M	SBP, DBP, TG, TC, LDL-C, HDL-C
			C	55 (24/31)	64.21 ± 6.12	4.92 ± 2.65		
Kong et al. (60)	2023	Hebei	C + BDJ	30 (30/0)	75.53 ± 3.92	2.74 ± 0.64	2W	SBP, DBP, PSQI
			C	30 (30/0)	75.47 ± 3.86	2.56 ± 0.56		
Lin et al. (61)	2017	Fujian	C + BDJ	58 (30/28)	60 ± 7.48	-	6M	SBP, DBP, HR
			C	58 (32/26)	60 ± 7.48	-		
Lin et al. (62)	2011	Anhui	C + WQX	68 (31/37)	60+	-	6M	SBP, DBP, HR
			C	59 (27/32)	60+	-		
He (63)	2015	Chongqing	C + BDJ	42 (22/20)	68.51 ± 2.97	8.23 ± 3.73	3M	SBP, DBP
			C	42 (23/19)	69.24 ± 2.45	8.51 ± 3.42		
Hu (64)	2022	Henan	C + TC	10 (−)	60-65		12W	SBP, DBP, TC, TG, LDL-C, HDL-C
			C	10 (−)	60-65			
Xie et al. (65)	2014	Shanxi	C + TC	25 (11/14)	60-70		12W	SBP, DBP
			C	25 (14/11)	60-70			
Cao et al. (66)	2024	Hebei	C + TC	40 (21/19)	66.98 ± 5.48	5.11 ± 2.21	3M	SBP, DBP, PSQI, SF-36
			C	40 (22/18)	67.02 ± 5.46	4.98 ± 2.23		
Sun et al. (67)	2014	Jiangsu	C + TC	38 (14/24)	68. 16 ± 4. 43		8W	SBP, DBP
			C	42 (24/18)	69. 10 ± 4. 28			
Wei et al. (68)	2015	Zhejiang	C + TC	42 (23/19)	72 ± 5.56	6 ± 5.46	6M	SBP, DBP
			C	42 (18/24)	70 ± 6.08	6 ± 5.46		
Li et al. (69)	2015	Heilongjiang	C + WQX	30 (−)	60-70		6M	SBP, DBP
			C	30 (−)	60-70			
Shen et al. (70)	2015	Jiangsu	C + WQX	31 (−)	60-80		6M	SBP, DBP, SF-36
			C + TC	40 (−)	60-80			
Li et al. (71)	2016	Henan	C + TC	120 (68/52)	62 ± 2.2		2M	SBP, DBP
			C		62 ± 2.2			
Lian et al. (72)	2020	Shanghai	C + BDJ	42 (15/27)	69.04 ± 9.69	16.25 ± 8.65	6M	SBP, DBP, SF-36
			C	42 (16/26)	70.71 ± 8.71	15.56 ± 8.39		
Chen et al. (73)	2020	Shanghai	C + BDJ	32 (12/20)	68.04 ± 9.69	65.71 ± 8.71	6M	SBP, DBP
			C	32 (11/21)	12.43 ± 9.71	11.50 ± 5.83		
Wu et al. (74)	2024	Shanxi	C + TC	30 (0/30)	61.73 ± 1.44		6M	SBP, DBP, HR
			C + WQX	30 (0/30)	62.27 ± 1.66			
			C	30 (0/30)	62.70 ± 1.62			
Lian et al. (75)	2024	Shanghai	C + BDJ	66 (30/36)	68.04 ± 9.69	12.43 ± 9.71	6M	SF-36, SBP, DBP
			C	66 (28/38)	65.71 ± 8.71	11.50 ± 5.83		
Yao (76)	2023	Hunan	C + BDJ	27 (13/14)	60-80	1–30	3M	PSQI, SBP, DBP
			C	30 (14/16)	60-80	1–30		
Wang (77)	2024	Jiangxi	C + TC	41 (18/23)	77.78 ± 7.93	18.51 ± 1.59	3M	SBP, DBP
			C	41 (18/23)	78.05 ± 7.92	17.41 ± 1.57		
Yang (78)	2018	Sichuan	C + TC	37 (16/21)	66.59 ± 4.53	12.14 ± 7.12	16W	SBP, DBP, HR, TC, TG, LDL-C, HDL-C, Du's Hypertension QOL Scale, PSQI
			C	38 (18/20)	67.47 ± 5.12	11.32 ± 4.48		

M, male; F, female; C, the control group; TShi, QG, Qigong; BDJ, Baduanjin; WQX, Wuqinxi; mos, mouths; WHO, World Health Organization; QOL, quality of Life; SF-36, medical outcomes study 36-item short form; SF-12, medical outcomes study 1-2item short form; PSQI, Pittsburgh sleep quality index; TC, total cholesterol; TG, triglyceride; LDL-C, low-density lipoprotein cholesterol; HDL-C, high-density lipoprotein cholesterol; HR, heart rate, SBP, systolic blood pressure; DBP, diastolic blood pressure.

### 3.2 Evaluation of risk of bias and GRADE assessment

**Figure 2:**
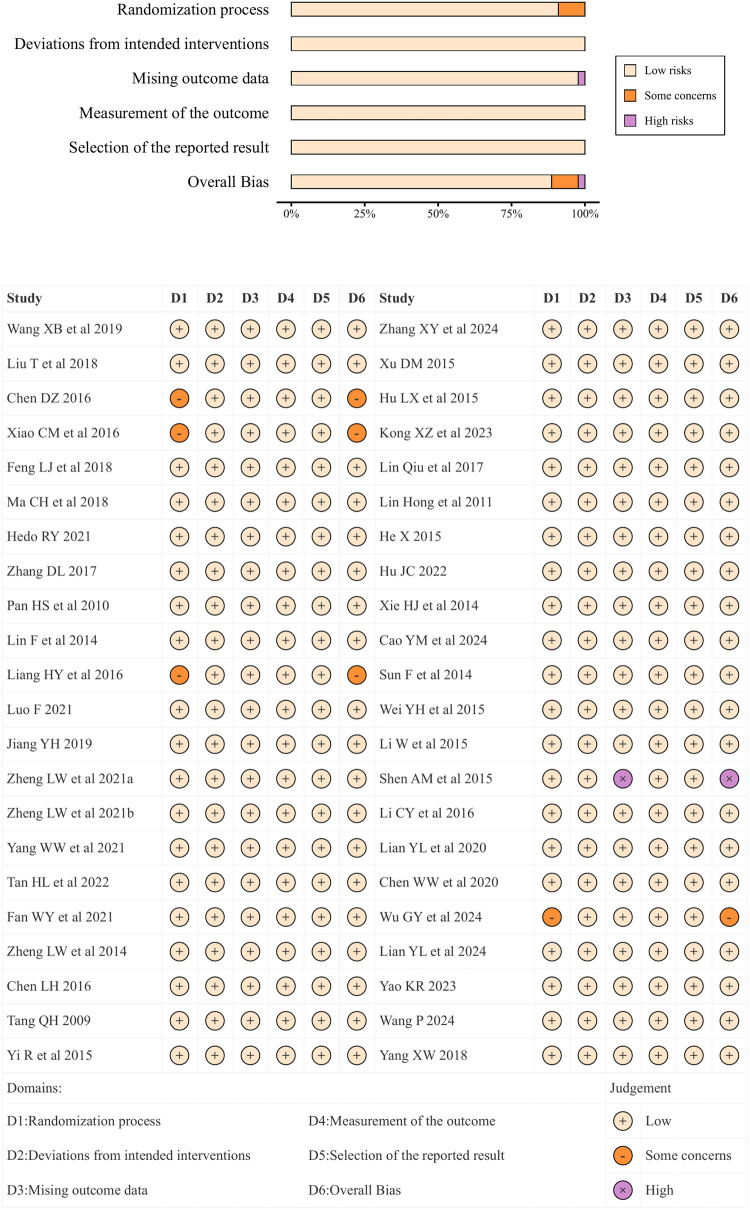
Evaluation of risk of bias in the included studies.

A total of 39 studies (88.6%) had low risk of bias, and four studies (9.1%) demonstrated some concerns. Four studies failed to report allocation concealment was performed or specific randomization approaches, and one study (2.3%) was judged to have a high risk of bias owing to more than 20% of missing data and the absence of intention-to-treat (ITT) analysis. Details are presented in Figure 2.

To assess the robustness of our findings, we performed an additional sensitivity analysis based on risk-of-bias judgments, excluding studies at moderate or high risk of bias and retaining only those at low risk of bias; we then recalculated SUCRA values. The results were consistent with those of the primary analysis (Supplementary Table S4).

Supplementary Table S2 presents the GRADE assessment results of the comparisons among groups. Most comparisons regarding SBP, DBP, HDL-C, QOL, and chest circumference showed medium to low levels of evidence, while most comparisons of total cholesterol, LDL-C, and HR indicated high to low levels, and most comparisons involving TG and PSQI demonstrated medium to high levels.

### 3.3 Results of NMA

Supplementary Figure S1 illustrates the proportion of contributions from direct evidence versus indirect evidence to the NMA results, as well as the number of the studies for each direct comparison.

Inconsistency for blood pressure (SBP and DBP) and HR was examined using loop-specific heterogeneity estimation, global inconsistency models, and node-splitting analysis (Supplementary Table S3). Loop-specific heterogeneity estimates indicated that all closed loops for SBP and HR showed good consistency, with inconsistency detected in only one closed loop for DBP. Global inconsistency testing indicated a *p* > 0.05 for both SBP and DBP, suggesting no significant global inconsistency in the network. For HR, however, *p* < 0.05 manifested significant global inconsistency in the network. Node-splitting analysis revealed no significant inconsistency (*p* > 0.05) between direct and indirect evidence for all comparisons of SBP and DBP, indicating reliable results. However, significant inconsistency (*p* < 0.05) between direct and indirect evidence was detected twice for HR. Since both the global inconsistency model (*p* < 0.05) and node-splitting analysis detected significant inconsistency for HR, the reliability of NMA-based indicators for HR was reduced.

**Figure 3:**
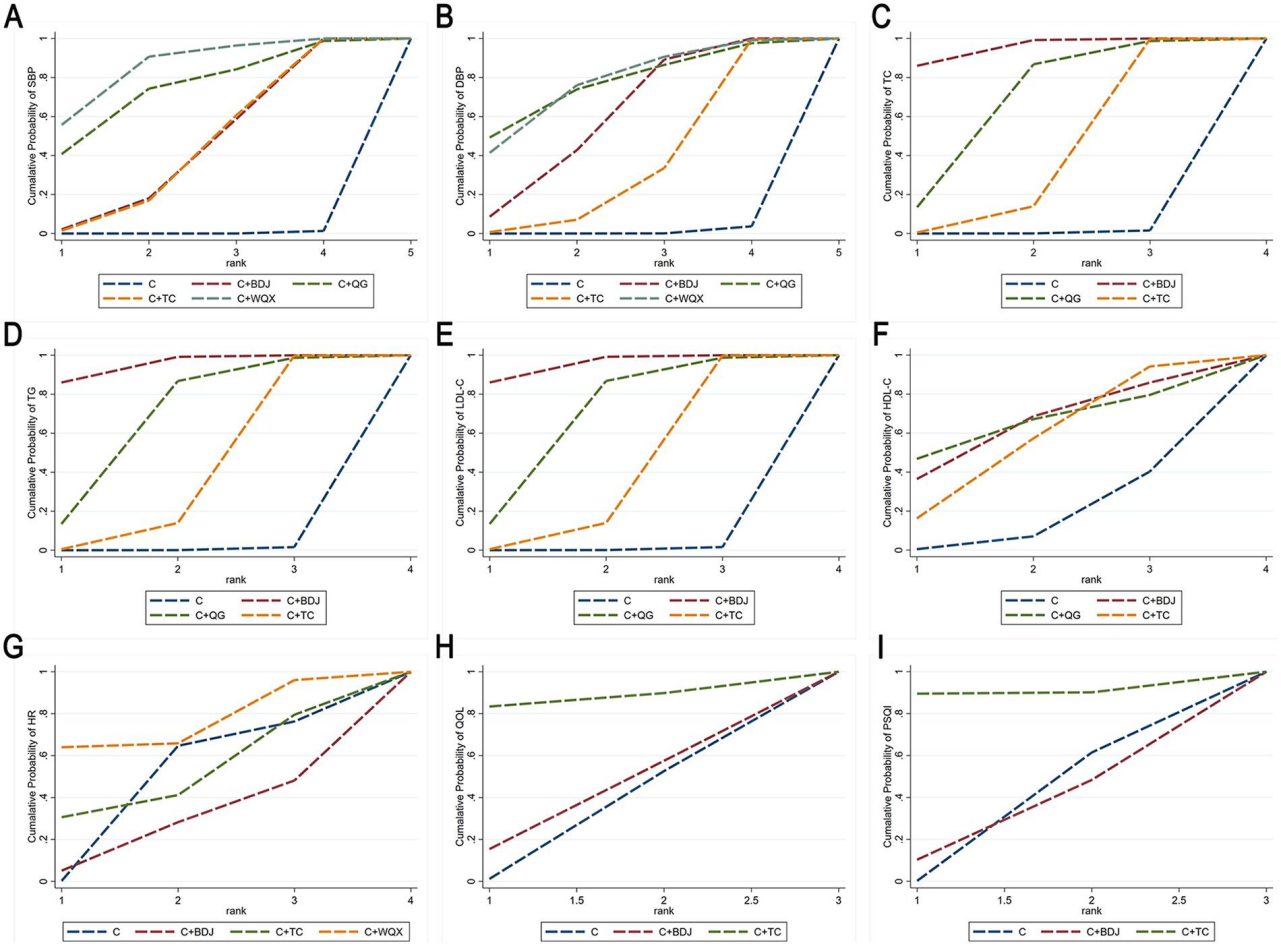
SUCRA for each intervention on SBP **(A)**, DBP **(B)**, TC **(C)**, TG **(D)**, LDL-C **(E)**, HDL-C **(F)**, QOL **(G)**, PSQI **(H)**, and HR **(I)**. SBP, systolic blood pressure; DBP, diastolic blood pressure; TC: total cholesterol; TG, triglyceride; LDL-C, low-density lipoprotein cholesterol; QOL, high-density lipoprotein cholesterol; QOL, quality of life; PSQI, Pittsburgh sleep quality index; HR, heart rate.

Supplementary Figure S2 presents forest plots with prediction intervals for blood pressure (SBP and DBP) and other outcomes. The forest plots report both the 95% CI and the 95% prediction interval (95% PrI) for the pooled effect sizes. The 95% CI reflects the precision of the effect estimation, while the 95% PrI predicts the possible range of results in future new studies. Figure 3 and Supplementary Table S4 display the SUCRA values for each intervention in the network for blood pressure (SBP and DBP) and secondary outcome indicators.

#### 3.3.1 Pooled estimates for blood pressure

**Figure 4:**
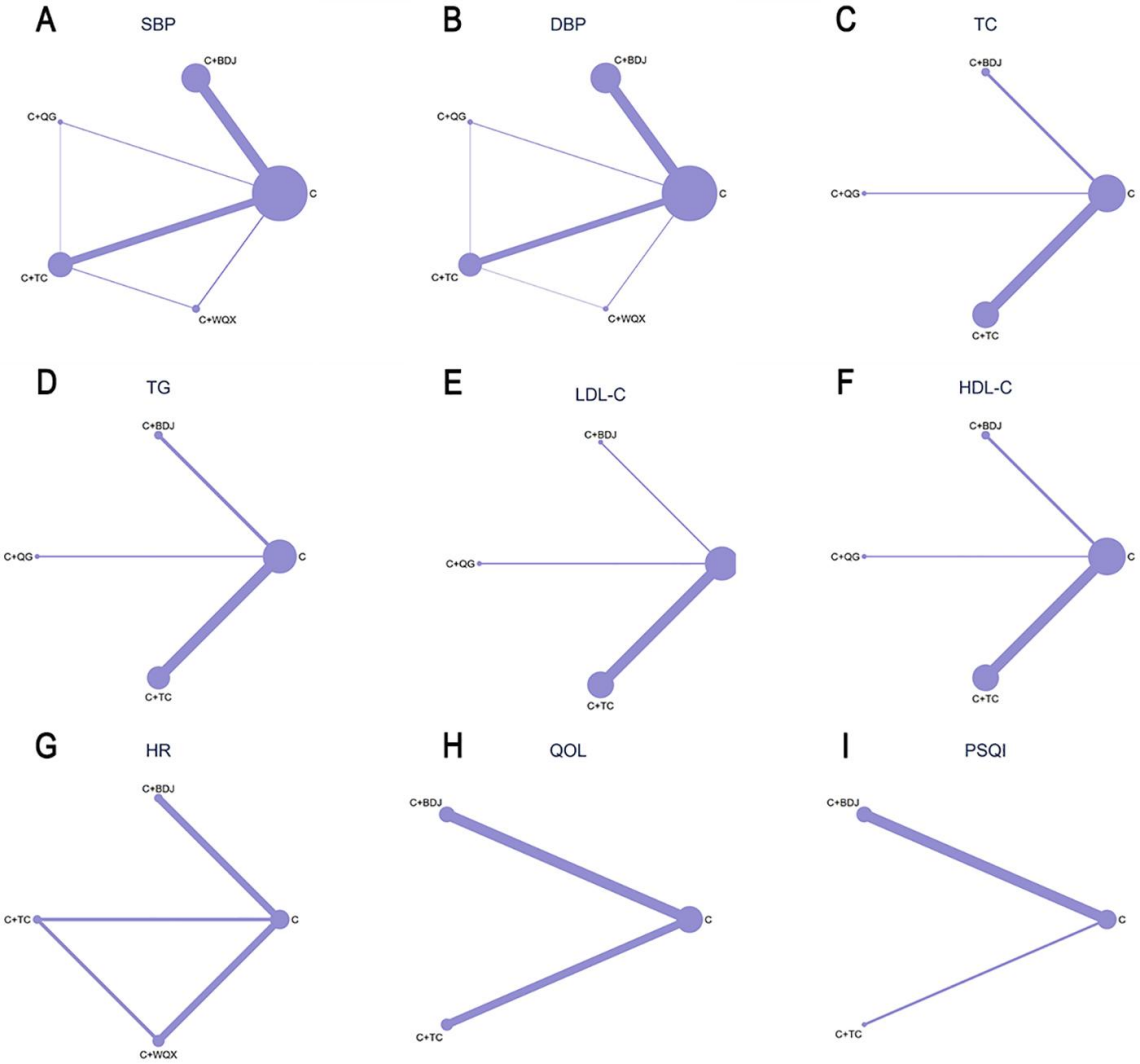
Network plot for the effects of traditional Chinese medicine exercises on blood pressure and secondary outcomes in older patients with hypertensive. **(A)** Systolic blood pressure; **(B)** diastolic blood pressure; **(C)** total cholesterol; **(D)** triglyceride; **(E)** low-density lipoprotein cholesterol; **(F)** high-density lipoproteincholesterol; **(G)** quality of life; **(H)** pittsburgh sleep quality index; **(I)** heart rate.

For SBP, four intervention regimens were included (*n* = 3,088). The network plot showed that the area of the node of C (the control group) was the largest, reflecting the largest size of the cumulative samples. The thickest line connected C + BDJ and C, suggesting the greatest amount of direct comparative evidence between these two groups (Figure 4A). NMA results showed that compared with C, C + TC [MD = −8.80 (−13.11, −4.54)], C + BDJ [MD = −8.78 (−12.44, −5.16)], C + QG [MD = −13.71 (−25.73, −1.76)], and C + WQX [MD = −15.23 (−23.61, −6.81)] significantly improved SBP (Table 3). SUCRA ranking results indicated that C + WQX (85.7%) was the most effective traditional Chinese medicine exercise for improving SBP, followed by C + QG (74.4%), C + BDJ (44.9%), C + TC (44.7%), and C (0.03%) (Figurec 3A).

**Table 3:** Network meta-analysis matrix of primary and secondary outcomes.

(A) SBP				
C	**−8.78** **(−12.44, −5.16)**	**−13.71** (**−25.73, −1.76)**	**−8.80** (**−13.11, −4.54)**	**−15.23** (**−23.61, −6.81)**
8.78 (5.16, 12.44)	C + BDJ	−4.93 (−17.47, 7.59)	−0.02 (−5.70, 5.63)	−6.45 (−15.59, 2.74)
13.71 (1.76, 25.73)	4.93 (−7.59, 17.47)	C + QG	4.92 (−7.45, 17.24)	−1.49 (−15.99, 12.96)
8.80 (4.54, 13.11)	0.02 (−5.63, 5.70)	−4.92 (−17.24, 7.45)	C + TC	−6.42 (−14.99, 2.24)
15.23 (6.81, 23.61)	6.45 (−2.74, 15.59)	1.49 (−12.96, 15.99)	6.42 (−2.24, 14.99)	C + WQX
(B) DBP				
C	**−6.22** (**−8.72, −3.72)**	**−8.86** (**−17.61, −0.06)**	**−4.15** (**−7.23, −1.10)**	**−8.41** (**−15.32, −1.57)**
6.22 (3.72, 8.71)	C + BDJ	−2.65 (−11.74, 6.49)	2.07 (−1.89, 6.02)	−2.19 (−9.54, 5.09)
8.86 (0.06, 17.61)	2.65 (−6.49, 11.7)	C + QG	4.72 (−4.36, 13.71)	0.45 (−10.69, 11.45)
4.15 (1.10, 7.23)	−2.066 (−6.02, 1.89)	−4.72 (−13.71, 4.36)	C + TC	−4.26 (−11.37, 2.81)
8.41 (1.57, 15.32)	2.19 (−5.09, 9.54)	−0.45 (−11.45, 10.69)	4.26 (−2.81, 11.37)	C + WQX
(C) TC				
C	**−1.16** (**−1.63, −0.71)**	**−0.80** (**−1.45, −0.15)**	**−0.46** (**−0.75, −0.20)**	
1.16 (0.71, 1.63)	C + BDJ	0.36 (−0.43, 1.17)	0.70 (0.15, 1.23)	
0.80 (0.15, 1.45)	−0.36 (−1.12, 0.43)	C + QG	0.34 (−0.39, 1.03)	
0.46 (0.20, 0.75)	−0.70 (−1.23, −0.15)	−0.34 (−1.03, 0.39)	C + TC	
(D) TG				
C	−0.44 (−0.91, 0.00)	−0.36 (−1.04, 0.32)	**−0.31** (**−0.60, −0.02)**	
0.44 (−0.00, 0.91)	C + BDJ	0.08 (−0.72, 0.91)	0.12 (−0.39, 0.68)	
0.36 (−0.32, 1.03)	−0.08 (−0.91, 0.72)	C + QG	0.04 (−0.69, 0.78)	
0.31 (0.02, 0.60)	−0.12 (−0.68, 0.39)	−0.04 (−0.78, 0.69)	C + TC	
(E) LDL−C				
C	**−1.38** (**−1.98, −0.79)**	**−0.85** (**−1.39, −0.31)**	**−0.35** (**−0.60, −0.17)**	
1.38 (0.79, 1.98)	C + BDJ	0.53 (−0.27, 1.33)	1.02 (0.36, 1.63)	
0.85 (0.31, 1.39)	−0.53 (−1.33, 0.27)	C + QG	0.49 (−0.12, 1.04)	
0.35 (0.17, 0.60)	−1.02 (−1.63, −0.36)	−0.49 (−1.04, 0.12)	C + TC	
(F) HDL-C				
C	0.20 (−0.24, 0.63)	0.23 (−0.40, 0.85)	0.13 (−0.07, 0.35)	
−0.20 (−0.63, 0.24)	C + BDJ	0.03 (−0.73, 0.80)	−0.07 (−0.54, 0.43)	
−0.23 (−0.85, 0.40)	−0.03 (−0.80, 0.73)	C + QG	−0.10 (−0.75, 0.58)	
−0.13 (−0.35, 0.07)	0.07 (−0.43, 0.54)	0.10 (−0.60, 0.75)	C + TC	
(G) HR				
C	−4.88 (−10.30, 0.11)	−1.48 (−8.8, 5.91)	−3.42 (−8.83, 1.80)	
4.87 (−0.11, 10.30)	C + BDJ	3.44 (−5.36, 12.62)	1.50 (−5.89, 9.01)	
1.48 (−5.91, 8.80)	−3.44 (−12.62, 5.36)	C + TC	−1.95 (−9.47, 5.36)	
3.42 (−1.8, 8.83)	−1.50 (−9.01, 5.89)	1.95 (−5.36, 9.47)	C + WQX	
(H) QOL				
C	9.57 (−4.71, 28.85)	9.09 (−8.70, 26.77)		
−9.57 (−28.85, 4.71)	C + BDJ	−0.47 (−27.40, 21.31)		
−9.09 (−26.77, 8.70)	0.47 (−21.31, 27.40)	C + TC		
(I) PSQI				
C	−2.42 (−4.24, −0.69)	−1.99 (−5.62, 1.61)		
**2.42** (**0.69, 4.24)**	C + BDJ	0.43 (−3.57, 4.50)		
1.99 (−1.61, 5.62)	−0.43 (−4.50, 3.57)	C + TC		

A, systolic blood pressure; B. diastolic blood pressure; C, total cholesterol; D, triglyceride; E, low-density lipoprotein cholesterol; F, high-density lipoproteincholesterol; G, heart rate; H, quality of life; I, Pittsburgh sleep quality index.

Bold values indicate statistical significance (*p* < 0.05).

As for DBP, four interventions in total were included (*n* = 2,998). The network plot showed that the area of the node of C was the largest, demonstrating the largest size of the cumulative samples. The thickest line connected C + BDJ and C, suggesting the greatest amount of direct comparative evidence between these two groups (Figure 4B). NMA results demonstrated that in comparison with C, C + TC [MD = −4.15 (−7.23, −1.10)], C + BDJ [MD = −6.22 (−8.72, −3.72)], C + QG [MD = −8.86 (−17.61, −0.06)], and C + WQX [MD = −8.41 (−15.32, −1.57)] significantly improved DBP (Table 3). SUCRA ranking results suggested that C + QG (76.8%) and C + WQX (76.8%) were the two most effective traditional Chinese medicine exercises for improving DBP, followed by C + BDJ (60.2%), C + TC (35.2%), and C (0.09%) (Figure 3B). Although SUCRA suggested a similar ranking pattern for DBP, several pairwise estimates were imprecise, the 95% confidence intervals (and reported prediction intervals) were relatively wide, and the absolute differences between interventions were small. Therefore, DBP rankings should be interpreted with caution.

#### 3.3.2 Pooled estimates for blood lipid

In terms of TC (total cholesterol), three intervention regimens were included (*n* = 647). The network plot showed that the area of the node C had was largest, reflecting the largest size of the cumulative samples. The thickest line connected C + TC (Tai Chi) and C, suggesting the greatest amount of direct comparative evidence between these two groups (Figure 4C). NMA results suggested that compared with C, C + TC [MD = −0.46 (−0.75, −0.20)], C + BDJ [MD = −1.16 (−1.63, −0.71)], and C + QG [MD = −0.80 (−1.45, −0.15)] significantly improved TC (Table 3). SUCRA ranking results showed that C + BDJ (95.1%) was the most effective traditional Chinese medicine exercise for improving total cholesterol, followed by C + QG (66.3%), C + TC (38.1%), and C (0.5%) (Figure 3C).

With regard to TG, three interventions were included (*n* = 647). The network plot demonstrated that the area of the node C was the largest, reflecting the largest size of the cumulative samples. The thickest line connected C + TC and C, suggesting the greatest amount of direct comparative evidence between these two groups (Figure 4D). NMA results showed that compared with C, only C + TC [MD = −0.31 (−0.60, −0.02)] significantly improved TG (Table 3). SUCRA ranking results showed that C + BDJ (95.1%) was the most effective traditional Chinese medicine exercise for improving TG, followed by C + QG (66.3%), C + TC (38.1%), and C (0.5%) (Figure 3D). Although SUCRA rankings reflect the probability that an intervention is among the best options, they may not align with statistical significance derived from effect estimates and confidence intervals. Therefore, when interpreting triglyceride rankings, both the magnitude and precision of the estimates should be considered, particularly in sparse networks with limited direct evidence.

For LDL-C, three intervention regimens in total were included (*n* = 599). The network plot indicated that the area of the node C was the largest, reflecting the largest size of the cumulative samples. The thickest line connected C + TC and C, suggesting the greatest amount of direct comparative evidence between these two groups (Figure 4E). NMA results showed that compared with C, C + QG [MD = −0.85 (−1.39, −0.31)], C + TC [MD = −0.35 (−0.60, −0.17)], and C + BDJ [MD = −1.38 (−1.98, −0.79)] significantly improved LDL-C (Table 3). Effectiveness rankings for improving LDL-C showed C + BDJ (SUCRA = 95.1%), C + QG (SUCRA = 66.3%), and C + TC (SUCRA = 38.1%), with all interventions superior to C (the control group) (SUCRA = 0.5%) (Figure 3E).

As for HDL-C, three intervention regimens were included (*n* = 647). The network plot demonstrated that the area of the node C was the largest, reflecting the largest size of the cumulative samples. The thickest line connected C + TC and C, suggesting the greatest amount of direct comparative evidence between these two groups (Figure 4F). NMA results showed that none of the pairwise comparisons between interventions reached statistical significance (Table 3). SUCRA ranking results showed that C + QG (64.5%) was the most effective traditional Chinese medicine exercise for improving HDL-C, followed by C + BDJ (63.7%), C + TC (55.9%), and C (15.9%) (Figure 3F).

#### 3.3.3 Pooled estimates for QOL and HR

For QOL, a total of two intervention regimens were included (*n* = 618). The network plot showed that the area of the node C was largest, reflecting the largest size of the cumulative samples. The thickest line connected C + BDJ and C, suggesting the greatest amount of direct comparative evidence between these two groups (Figure 4G). NMA results showed that in comparison with the control group (C), none of the interventions demonstrated statistical significance (Table 3). SUCRA ranking results showed that C + TC (78.6%) was the most effective traditional Chinese medicine exercise for improving QOL, followed by C + BDJ (72.5%) and C (8.9%) (Figure 3G).

Regarding PSQI, two intervention regimens were included (*n* = 344). The network plot showed that the area of the node C was the largest, signifying the largest size of the cumulative samples. The thickest line connected C + BDJ and C, suggesting the greatest amount of direct comparative evidence between these two groups (Figure 4H). NMA results showed that compared with C, only C + BDJ [MD = −2.42 (−4.24, −0.69)] significantly improved PSQI (Table 3). The effectiveness ranking for improving PSQI was C + BDJ (SUCRA = 80.5%) and C + TC (SUCRA = 64.1%), with both interventions superior to group C (SUCRA = 5.4%) (Figure 3H).

In terms of HR, four intervention regimens in total were included (*n* = 401). The network plot showed that the area of the node of C was the largest, demonstrating the largest size of the cumulative samples. The thickest lines connected C + TC with C and C + TC with C + BDJ, suggesting the greatest amount of direct comparative evidence for these two groups (Figure 4I). NMA results indicated that none of the pairwise comparisons between interventions achieved statistical significance (Table 3). SUCRA ranking results showed that C + BDJ (83.4%) was the most effective traditional Chinese medicine exercise for improving HR, followed by C + WQX (65.1%), C + TC (37.6%), and C (13.9%) (Figure 3I).

### 3.4 Publication bias

**Figure 5:**
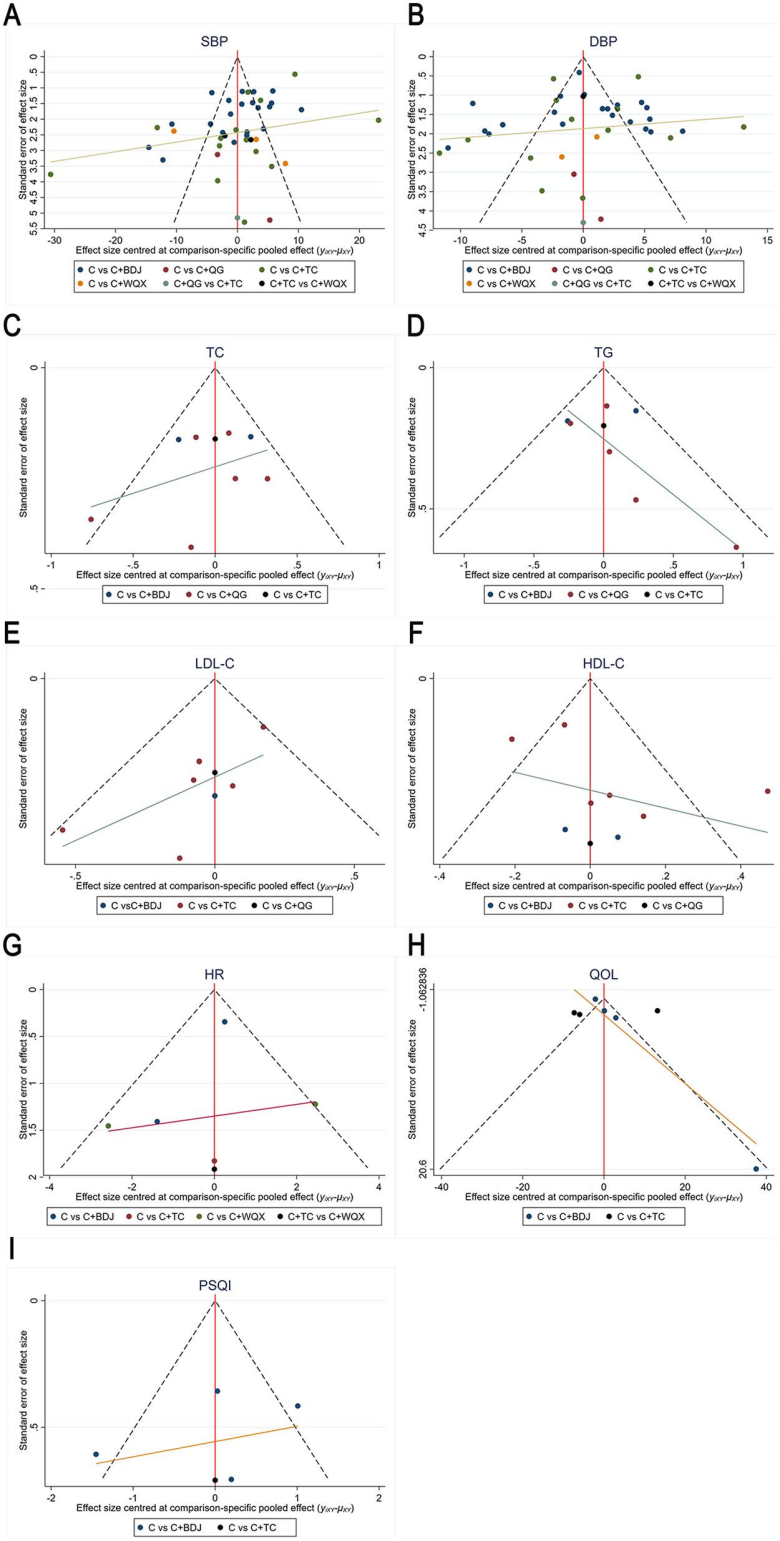
Funnel plot for SBP **(A)**, DBP **(B)**, total cholesterol **(C)**, TG **(D)**, LDL-C **(E)**, HDL-C **(F)**, QOL **(G)**, PSQI **(H)**, and HR **(I)**. A: C, B: C + BDJ, C: C + QG, D: C + TC, E: C + WQX. SBP, systolic blood pressure; DBP, diastolic blood pressure; TC, total cholesterol; TG, triglyceride; LDL-C, low-density lipoprotein cholesterol; QOL, high-density lipoprotein cholesterol; QOL, quality of life; PSQI, Pittsburgh sleep quality index; HR, heart rate.

The funnel plot indicated that all the included studies were distributed on both sides of the central line (Figure 5). The result of Egger's test for SBP (*p* = 0.0007) suggested that there might be significant publication bias (*p* < 0.05). DBP (*p* = 0.6471), total cholesterol (*p* = 0.5560), TG (*p* = 0.6931), and HDL-C (*p* = 0.1048) indicated that no significant publication bias was observed (Supplementary Table S5).

## 4 Discussion

This systematic review and NMA included 44 RCTs comparing the effects of different traditional Chinese medicine exercises on SBP, DBP, total cholesterol, TG, LDL-C, HDL-C, HR, QOL, and PSQI in older patients with hypertension. The NMA results demonstrated that compared with C (the control group), C + TC, C + QG, C + BDJ, and C + WQX improved SBP and DBP among older patients with hypertension. C + BDJ, C + TC, and C + QG all significantly improved total cholesterol and LDL-C in older patients suffering from hypertension. C + TC improved TG, and C + BDJ improved PSQI among older patients with hypertension. SUCRA ranking results indicated that C + BDJ and C + WQX had the greatest potential to become the two best interventions in improving SBP, DBP, total cholesterol, TG, LDL-C, HR, QOL, and PSQI among older patients with hypertension.

This study revealed that blood pressure indicators (such as SBP and DBP) in older patients with hypertension were significantly improved by traditional Chinese medicine exercises. In this study, WQX and QG performed well, with reductions in SBP and DBP ranking highest among the traditional Chinese medicine exercise therapies. In particular, WXQ and QG significantly reduced DBP, and were tied for the best therapy in SUCRA ranking analysis. These findings are in line with those of Zhang et al. (28), who found WQX to be the most effective exercise therapy for reducing DBP. Although the ranking results indicated that QG was also one of the most effective interventions for reducing DBP, this result should be interpreted cautiously, as only two studies were included. Therefore, further high-quality and large-scale RCTs are needed to confirm this conclusion. In addition, compared with previous studies, our findings differ in terms of the indicators for SBP. For instance, Zhang et al. (28) found TC to be superior to WQX in reducing SBP, while in this study, WXQ was superior to TC in reducing SBP. This discrepancy may be due to the differences in the characteristics of the samples, the duration of interventions, and the intensity of exercises. Under these circumstances, WQX is considered to be the most effective exercise therapy for lowering the blood pressure levels of older patients suffering from hypertension. From a mechanism perspective, traditional Chinese medicine exercise may regulate blood pressure through multiple mechanisms, including improving the inflammatory state, oxidative stress, and endothelial dysfunction among individuals suffering from hypertension. The proposed mechanisms are as follows: (1) After exercise, reductions in blood pressure were associated with increased antioxidant enzyme activity, decreased free radical production, and improved oxidative stress status (29). (2) Blood pressure is lowered by reducing oxidative stress, increasing cardiac output and vascular shear stress, and upregulating the phosphorylation of eNOS as well as the activity of related enzymes, thereby enhancing the bioavailability of nitric oxide (30). (3) Blood pressure decreases by reducing inflammatory factors, such as C-reactive protein (31).

This research found that engaging in traditional Chinese medicine exercises significantly improves blood lipid levels among patients. Consistent with prior findings (32), our conclusion was not different: TC decreased total cholesterol, TG, and LDL-C. This study also revealed that BDJ more significantly improves total cholesterol and LDL-C compared to other exercise therapies. This may be because BDJ, as a popular physical and mental exercise, is unique and easier to learn than other physical and mental exercises such as Tai Chi (33), thereby more effectively promoting lipid metabolism in skeletal muscles (34).

Although evidence for lipid outcomes is less extensive than that for blood pressure, TCM exercise therapies may still confer clinically meaningful cardiometabolic benefits in older adults with hypertension. In our analysis, Baduanjin was associated with reductions in total cholesterol (−1.16 mmol/L) and LDL-C (−1.38 mmol/L), while Tai Chi was associated with a reduction in triglycerides (−0.31 mmol/L); however, no consistent improvement was observed for HDL-C (Table 3). Large-scale evidence indicates that the magnitude of LDL-C reduction is approximately log-linearly associated with a lower risk of atherosclerotic cardiovascular disease (ASCVD). The cholesterol treatment trialists' (CTT) Collaboration reported that each 1 mmol/L reduction in LDL-C reduces the relative risk of major vascular events by slightly more than one-fifth. Although exercise and statins act through different mechanisms, this relationship provides a useful benchmark for interpreting the potential clinical relevance of the observed lipid-lowering effects (79). Consistent with European Society of Cardiology/European Atherosclerosis Society (ESC/EAS) guidelines, which prioritize LDL-C as the primary therapeutic target and recommend lifestyle modification as the foundation of lipid management, these exercise therapies may offer practical adjunctive value for patients unable to sustain high-intensity exercise long term (80). Nevertheless, given the small sample size of the lipid network, the relatively short intervention duration (∼12 weeks), and prior evidence suggesting modest and heterogeneous exercise-related lipid responses in older adults with hypertension, these findings should be interpreted cautiously and require confirmation in larger, longer-term randomized controlled trials using standardized lipid measures and clinically relevant endpoints (81).

In terms of improving PSQI, only BDJ has been proven to be effective. Multiple studies have shown that PSQI significantly increased after BDJ interventions. In a 12-week BDJ exercise program for older patients with stroke, significant improvement in PSQI was observed (35).

This NMA suggests that TCM exercise therapies may be selected according to the predominant clinical needs of older adults with hypertension. When blood pressure reduction is the primary goal, Wuqinxi and Qigong appear to offer greater improvements in SBP/DBP. For patients in whom dyslipidemia is the main concern, Baduanjin may be more effective for reducing total cholesterol and LDL-C, whereas Tai Chi may be more effective for lowering triglycerides. Notably, Baduanjin was the only intervention significantly associated with improved sleep quality, as measured by PSQI, suggesting that it may be a preferred option for patients with comorbid insomnia or poor sleep quality.

Compared with previous studies, this research offers several advantages. First, a comprehensive meta-analysis was conducted of common traditional Chinese medicine exercise therapies, systematically assessing and comparing the efficacy of each therapy to determine the optimal exercise. Second, to ensure the comprehensiveness of the meta-analysis and the richness of the data, extensive literature searches were carried out across eight databases. The included studies were all relevant to this study and had no time restrictions. However, several limitations must be acknowledged. First, the included studies were mainly conducted in China, which might affect the generalizability of the results. For example, traditional Chinese medicine exercises, comprising Tai Chi and Baduanjin, have long histories and high recognition in China, leading to greater compliance during implementation. However, in other cultural contexts, differences in perception may affect patients' compliance. Therefore, to enhance the generalizability of the results of this research, future studies need to include more diverse samples. Second, because of the limited number of included studies and the heterogeneity of the exercise interventions, the time–dose responses of different traditional Chinese medicine exercises were not explored. Third, four included studies were rated as demonstrating a medium risk of bias, and one included study showed a high risk of bias, which, to some extent, may have affected overall study quality. Fourth, indicators for SBP suggested significant publication bias, which might be attributed to the small sample sizes and the influence of publication bias on the results. Fifth, although all included studies applied a consistent hypertension threshold (≥140/90 mmHg; Supplementary Table S6), differences across guideline versions—and in blood pressure measurement and diagnostic confirmation procedures—may have introduced residual diagnostic misclassification, thereby contributing to heterogeneity. Sixth, although all trials enrolled participants aged ≥60 years, reported age ranges and distributions (e.g., 60–65, 60–70, 60–80, or “60+”) varied, which may have increased clinical heterogeneity and limited the comparability and generalizability of the pooled estimates. Seventh, the duration of TCM exercise interventions ranged from 2 to 26 weeks (mean ≈17 weeks), with most lasting 12 weeks. Such variation in intervention length may influence the stability of effect estimates and SUCRA rankings. Eighth, even within a single modality (e.g., Tai Chi), differences in style or form (e.g., simplified 24-form), training frequency, and session duration may also contribute to heterogeneity. Ninth, quality-of-life outcomes were assessed using different instruments (e.g., SF-36, SF-12, and the Duchenne Hypertension Quality of Life Scale). Although we used SMDs to pool results across scales, instrument differences may still affect comparability and interpretation. Tenth, blinding of participants and interventionists is often infeasible in exercise trials, which may bias effect estimates through performance effects or outcome assessment bias. Accordingly, SUCRA-based treatment rankings should be interpreted with caution.

## 5 Conclusion

This study demonstrated that different traditional Chinese medicine exercises have distinct and unique effects on improving blood pressure levels and lipid profiles. In particular, WQX and QG were effective in lowering blood pressure, while BDJ showed results in improving PSQI and lipid profiles (total cholesterol and LDL-C). TC demonstrated significant therapeutic effects in terms of improving total cholesterol. Nevertheless, all exercise therapies failed to significantly improve HR, HDL-C, and QOL. Given the limitations of the research—including its concentration in China, heterogeneity of exercise interventions, the small sample size, and quality issues—future studies need to focus on investigating whether traditional Chinese exercise therapies can effectively improve blood pressure levels and other related outcomes for populations outside Asia.

## Data Availability

The original contributions presented in the study are included in the article/Supplementary Material, further inquiries can be directed to the corresponding author.

## Supplementary material

Supplementary Table S1 Search strategy of PubMed, Embase, Cochrane, Web of Science, CNKI, WanFang, VIP, and CBM.

Supplementary Table S2 GRADE for the primary and secondary outcomes.

Supplementary Table S3 Inconsistency of SBP, DBP, and HR tested by loop-specific heterogeneity estimates, inconsistency model and node splitting analysis.

Supplementary Table S4 Ranking of exercise interventions in order of effectiveness.

Supplementary Table S5 Egger’s test for the primary and secondary outcomes.

Supplementary Figure S1 Contributions of direct and indirect comparisons to NMA and the number of studies of each direct comparison. **(A)** Systolic blood pressure; **(B)** diastolic blood pressure; **(C)** total cholesterol; **(D)** triglyceride; **(E)** low-density lipoprotein cholesterol; **(F)** high-density lipoproteincholesterol; **(G)** quality of life; **(H)** Pittsburgh sleep quality index; and **(I)** heart rate.

Supplementary Figure S2 Forest plots of eligible comparisons. **(A)** Systolic blood pressure; **(B)** diastolic blood pressure; **(C)** total cholesterol; **(D)** triglyceride; **(E)** low-density lipoprotein cholesterol; **(F)** high-density lipoproteincholesterol; **(G)** Pittsburgh sleep quality index; and **(H)** quality of life.

Supplementary Figure S3 Network map for the primary and secondary outcomes.
