# Supplementary material for: Comparison of the therapeutic effects of traditional Chinese medicine exercise therapies on blood pressure, lipids, and sleep quality among older patients suffering from hypertension: a systematic review and network meta-analysis
Source: Front Cardiovasc Med. 2026 Mar 11;13:1707525. doi: 10.3389/fcvm.2026.1707525 (PMC13013410; doi:10.3389/fcvm.2026.1707525)
Supplement: Supplementary Table S1 — Search strategy of PubMed, Embase, Cochrane, Web of Science, CNKI, WanFang, VIP, and CBM. [file Datasheet1.docx]

**Supplementary Materials**

**Table S1.** Search strategy of PubMed, Embase, Cochrane, Web of Science, CNKI, WanFang, VIP, and CBM.

**China Biology Medicine disc (CBM)**

| **No.** | **Query** | **Results** |
| --- | --- | --- |
| 1) | "Hypertension "[Common field: intelligent] | 952742 |
| 2) | "Tai Chi "[common field: intelligence] OR" Dacheng Quan "[common field: intelligence] OR "Yi Quan "[common field: intelligence] OR" Baduanjin "[common field: intelligence] OR "Wuqinxi "[common field: intelligence] OR" Qigong "[common field: intelligence] OR "Yi Jin Jing "[common field: intelligence] OR "Six-character formula "[Common field: Intelligence] | 32324 |
| 3) | "Random "[Common field: intelligent] | 2348595 |
| 4) | (" random "[common fields: intelligent]) AND ((" tai chi" [common fields: intelligent] OR "dacheng boxing" [common fields: intelligent] OR "fist" [common fields: intelligent] OR "eight brocade" [common fields: intelligent] OR "wuqinxi" [common fields: intelligent] the OR "Qigong" [common fields: intelligent] OR "yi jin jing" [common fields: intelligent] OR "six tactic" [common fields: intelligent]) AND (" hypertension "[common fields: intelligent])) | 139 |

**VIP**

| **No.** | **Query** | **Results** |
| --- | --- | --- |
| 1) | "Hypertension "[Arbitrary field] | 198,634 |
| 2) | "Tai chi" [arbitrary field] + "dacheng boxing" [arbitrary field] + "fist" [arbitrary field] + "eight brocade" [field] any + "wuqinxi" [arbitrary field] + "qigong" [arbitrary field] + "yi jin jing" [arbitrary field] + "six tactic" [the] any field | 33,406 |
| 3) | "Random "[any field] | 2,569,055 |
| 4) | (" random "[] any field) AND ((" tai chi" [arbitrary field] + "dacheng boxing" [arbitrary field] + "fist" [arbitrary field] + "eight brocade" [arbitrary field] + "wuqinxi" [arbitrary field] + "qigong" [arbitrary field] + "yi jin jing" [arbitrary field] + "six tactic" [] any field) AND (" Hypertension "[any field]) | 78 |

**Chinese National Knowledge Infrastructure(CNKI)**

| **No.** | **Query** | **Results** |
| --- | --- | --- |
| 1) | "Hypertension "[Abstract] | 462,926 |
| 2) | "Tai chi" [piece clearance pick] + "dacheng boxing" [piece clearance pick] + "fist" [piece clearance pick] + "eight brocade" [piece clearance pick] + "wuqinxi" [piece clearance pick] + "qigong" [piece clearance pick] + "yi jin jing" [piece clearance pick] + "six tactic" [article close to pick] | 35,425 |
| 3) | "Random "[Abstract] | 2,618,358 |
| 4) | (" random "[article close pick]) AND ((" tai chi" [piece clearance pick] + "dacheng boxing" [piece clearance pick] + "fist" [piece clearance pick] + "eight brocade" [piece clearance pick] + "wuqinxi" [piece clearance pick] + "qigong" [piece clearance pick] + "yi jin jing" [piece clearance pick] + "six tactic" [shut] picked article) AND (" High blood pressure ") | 163 |

**Wanfang**

| **No.** | **Query** | **Results** |
| --- | --- | --- |
| 1) | "High Blood pressure "[all] | 708,732 |
| 2) | "Tai chi" [all] OR "dacheng boxing" [all] OR [all] "fist" OR "eight brocade" [all] OR "wuqinxi" [all] OR [all] "qigong" OR "yi jin jing" [all] OR "six tactic" [all] | 63,442 |
| 3) | "Random "[all] | 3,516,345 |
| 4) | (" random "[all]) AND ((" tai chi" [all] OR "dacheng boxing" [all] OR [all] "fist" OR "eight jin [all]" OR "wuqinxi" [all] OR [all] "qigong" OR "yi jin jing" [all] OR "six tactic" [all]) AND (" High blood pressure "[all]) | 157 |

**Embase**

| **No.** | **Query** | **Results** |
| --- | --- | --- |
| #1 | 'hypertension'/exp | 1140688 |
| #2 | 'high blood pressure*':ti,ab,kw OR 'htn':ti,ab,kw OR 'hypertension':ti,ab,kw OR 'hypertensive disease':ti,ab,kw OR 'hypertensive effect':ti,ab,kw OR 'hypertensive reaction':ti,ab,kw OR 'hypertensive response':ti,ab,kw | 844495 |
| #3 | #1 OR #2 | 1391435 |
| #4 | 'tai chi'/exp OR 'qigong'/exp | 5462 |
| #5 | 'changing tendon exercise':ti,ab,kw OR 'chi kung':ti,ab,kw OR 'ch i kung':ti,ab,kw OR 'chigung':ti,ab,kw OR 'dacheng quan':ti,ab,kw OR 'eight trigrams boxing':ti,ab,kw OR 'five animal boxing':ti,ab,kw OR 'five animal exercises':ti,ab,kw OR 'five mimic animal exercise':ti,ab,kw OR 'hexagram boxing':ti,ab,kw OR 'qi gong':ti,ab,kw OR 'qigong':ti,ab,kw OR 'shadow boxing':ti,ab,kw OR 'shadowboxing':ti,ab,kw OR 'six character formula':ti,ab,kw OR 'six words':ti,ab,kw OR 'tai chi':ti,ab,kw OR 't ai chi':ti,ab,kw OR 'tai ji':ti,ab,kw OR 'taiji':ti,ab,kw OR 'taijiquan':ti,ab,kw OR 'tendon change classic':ti,ab,kw OR 'will boxing':ti,ab,kw OR 'yi jin jing':ti,ab,kw OR 'yi jinjing':ti,ab,kw OR 'yijin jing':ti,ab,kw OR 'yiquan':ti,ab,kw OR 'eight sectioned exercise':ti,ab,kw OR 'baduanjin':ti,ab,kw OR 'ba duan jin':ti,ab,kw OR 'eight section brocade':ti,ab,kw | 5774 |
| #6 | #4 OR #5 | 7130 |
| #7 | #3 AND #6 | 394 |

**Pubmed**

| **Search number** | **Search Details** | **Results** |
| --- | --- | --- |
| 1 | "Hypertension"[MeSH Terms] | 331,470 |
| 2 | "high blood pressure*"[Title/Abstract] OR "HTN"[Title/Abstract] OR "Hypertension"[Title/Abstract] OR "hypertensive disease"[Title/Abstract] OR "hypertensive effect"[Title/Abstract] OR "hypertensive reaction"[Title/Abstract] OR "hypertensive response"[Title/Abstract] OR "Hypertension"[MeSH Terms] | 604,902 |
| 3 | "Tai Ji"[MeSH Terms] OR "Qigong"[MeSH Terms] | 1,959 |
| 4 | "chi kung"[Title/Abstract] OR "Ch i Kung"[Title/Abstract] OR "chigung"[Title/Abstract] OR "eight trigrams boxing"[Title/Abstract] OR "qi gong"[Title/Abstract] OR "Qigong"[Title/Abstract] OR "shadow boxing"[Title/Abstract] OR "Shadowboxing"[Title/Abstract] OR "six character formula"[Title/Abstract] OR "Six Words"[Title/Abstract] OR "Tai Chi"[Title/Abstract] OR "T ai Chi"[Title/Abstract] OR "Tai Ji"[Title/Abstract] OR "Taiji"[Title/Abstract] OR "Taijiquan"[Title/Abstract] OR "Yi jin jing"[Title/Abstract] OR "Yi jinjing"[Title/Abstract] OR "Yijin Jing"[Title/Abstract] OR "Yiquan"[Title/Abstract] OR "Baduanjin"[Title/Abstract] OR "Ba Duan Jin"[Title/Abstract] OR "eight section brocade"[Title/Abstract] OR "Tai Ji"[MeSH Terms] OR "Qigong"[MeSH Terms] | 4,327 |
| 5 | ("high blood pressure*"[Title/Abstract] OR "HTN"[Title/Abstract] OR "Hypertension"[Title/Abstract] OR "hypertensive disease"[Title/Abstract] OR "hypertensive effect"[Title/Abstract] OR "hypertensive reaction"[Title/Abstract] OR "hypertensive response"[Title/Abstract] OR "Hypertension"[MeSH Terms]) AND ("chi kung"[Title/Abstract] OR "Ch i Kung"[Title/Abstract] OR "chigung"[Title/Abstract] OR "eight trigrams boxing"[Title/Abstract] OR "qi gong"[Title/Abstract] OR "Qigong"[Title/Abstract] OR "shadow boxing"[Title/Abstract] OR "Shadowboxing"[Title/Abstract] OR "six character formula"[Title/Abstract] OR "Six Words"[Title/Abstract] OR "Tai Chi"[Title/Abstract] OR "T ai Chi"[Title/Abstract] OR "Tai Ji"[Title/Abstract] OR "Taiji"[Title/Abstract] OR "Taijiquan"[Title/Abstract] OR "Yi jin jing"[Title/Abstract] OR "Yi jinjing"[Title/Abstract] OR "Yijin Jing"[Title/Abstract] OR "Yiquan"[Title/Abstract] OR "Baduanjin"[Title/Abstract] OR "Ba Duan Jin"[Title/Abstract] OR "eight section brocade"[Title/Abstract] OR ("Tai Ji"[MeSH Terms] OR "Qigong"[MeSH Terms])) | 181 |

**Web of Science**

| **#** | **Search Query** | **Results** |
| --- | --- | --- |
| 1 | TS=((high blood pressure*) OR (HTN) OR (hypertension) OR (hypertensive disease) OR (hypertensive effect) OR (hypertensive reaction) OR (hypertensive response)) | 556299 |
| 2 | TS=((changing tendon exercise) OR (chi kung) OR (Ch i Kung) OR (chigung) OR (Dacheng Quan) OR (eight trigrams boxing) OR (five animal boxing) OR (five animal exercises) OR (five mimic animal exercise) OR (hexagram boxing) OR (qi gong) OR (Qigong) OR (shadow boxing) OR (Shadowboxing) OR (six character formula) OR (Six Words) OR (Tai Chi) OR (T ai Chi) OR (Tai Ji) OR (Taiji) OR (Taijiquan) OR (Tendon Change Classic) OR (will boxing) OR (Yi jin jing) OR (Yi jinjing) OR (Yijin Jing) OR (Yiquan) OR (eight sectioned exercise) OR (Baduanjin ) OR (Ba Duan Jin) OR (eight section brocade)) | 33750 |
| 3 | #2 AND #1 | 513 |

**Cochrane**

| **ID** | **Search** | **Hits** |
| --- | --- | --- |
| #1 | MeSH descriptor: [Hypertension] explode all trees | 25159 |
| #2 | ('high blood pressure*' OR 'HTN' OR 'hypertension' OR 'hypertensive disease' OR 'hypertensive effect' OR 'hypertensive reaction' OR 'hypertensive response'):ti,ab,kw | 101010 |
| #3 | #1 OR #2 | 101011 |
| #4 | MeSH descriptor: [Tai Ji] explode all trees | 587 |
| #5 | MeSH descriptor: [Qigong] explode all trees | 172 |
| #6 | ('changing tendon exercise' OR 'chi kung' OR 'Ch i Kung' OR 'chigung' OR 'Dacheng Quan' OR 'eight trigrams boxing' OR 'five animal boxing' OR 'five animal exercises' OR 'five mimic animal exercise' OR 'hexagram boxing' OR 'qi gong' OR 'Qigong' OR 'shadow boxing' OR 'Shadowboxing' OR 'six character formula' OR 'Six Words' OR 'Tai Chi' OR 'T ai Chi' OR 'Tai Ji' OR 'Taiji' OR 'Taijiquan' OR 'Tendon Change Classic' OR 'will boxing' OR 'Yi jin jing' OR 'Yi jinjing' OR 'Yijin Jing' OR 'Yiquan' OR 'eight sectioned exercise' OR 'Baduanjin' OR 'Ba Duan Jin' OR 'eight section brocade'):ti,ab,kw | 4969 |
| #7 | #4 OR #5 OR #6 | 4969 |
| #8 | #3 AND #7 | 319 |

**Table S2.** GRADE for the primary and secondary outcomes.

**SBP**

| **Comparison** | **Direct evidence** | | **Indirect evidence** | | **Network Meta-Analysis** | |
| --- | --- | --- | --- | --- | --- | --- |
|  | **MD[95%CI]** | **Certainty of evidence** | **MD[95%CI]** | **Certainty of evidence** | **MD[95%CI]** | **Certainty of evidence** |
| C+BDJ vs C | -8.09 [-10.22, -5.96] | **⨁⨁**OO | - | - | -8.09 [-10.22, -5.96] | **⨁⨁**OO |
| C+BDJ vs C+QG | - | - | 9.60[3.39,15.81] | **⨁⨁**OO | 9.60[3.39,15.81] | **⨁⨁**OO |
| C+BDJ vs C+TC | - | - | 0.67[3.2,-1.86] | **⨁⨁**OO | 0.67[3.2,-1.86] | **⨁⨁**OO |
| C+BDJ vs C+WQX | - | - | 9.97[18.85,1.09] | **⨁⨁**OO | 9.97[18.85,1.09] | **⨁⨁**OO |
| C+QG vs C | -17.69 [-21.77, -13.61] | **⨁⨁**OO | - | - | -17.69 [-21.77, -13.61] | **⨁⨁**OO |
| C+QG vs C+TC | -1.72 [-8.37, -11.88] | **⨁⨁⨁**O | - | - | -1.72 [-8.37, -11.88] | **⨁⨁⨁**O |
| C+QG vs C+WQX | - | - | 0.37[7.3,-6.56] | **⨁⨁**OO | 0.37[7.3,-6.56] | **⨁⨁**OO |
| C+TC vs C | -8.76 [-13.42, -4.10] | **⨁⨁**OO | - | - | -8.76 [-13.42, -4.10] | **⨁⨁**OO |
| C+TC vs C+WQX | 2.30 [-2.90, 7.50] | **⨁⨁⨁**O | - | - | 2.30 [-2.90, 7.50] | **⨁⨁⨁**O |
| C+WQX vs C | -18.06 [-29.07, -7.05] | **⨁⨁**OO | - | - | -18.06 [-29.07, -7.05] | **⨁⨁**OO |

**DBP**

| **Comparison** | **Direct evidence** | | **Indirect evidence** | | **Network Meta-Analysis** | |
| --- | --- | --- | --- | --- | --- | --- |
|  | **MD[95%CI]** | **Certainty of evidence** | **MD[95%CI]** | **Certainty of evidence** | **MD[95%CI]** | **Certainty of evidence** |
| C+BDJ vs C | -6.05 [-6.56, -5.55] | **⨁⨁**OO | - | - | -6.05 [-6.56, -5.55] | **⨁⨁**OO |
| C+BDJ vs C+QG | - | - | 6.77 [11.67, 1.87] | **⨁⨁**OO | 6.77 [11.67, 1.87] | **⨁⨁**OO |
| C+BDJ vs C+TC | - | - | ‘-2.34 [-0.07, -4.61] | **⨁⨁**OO | ‘-2.34 [-0.07, -4.61] | **⨁⨁**OO |
| C+BDJ vs C+WQX | - | - | 4.66 [7.34, 1.98] | **⨁⨁**OO | 4.66 [7.34, 1.98] | **⨁⨁**OO |
| C+QG vs C | -12.82 [-18.23, -7.42] | **⨁⨁**OO | - | - | -12.82 [-18.23, -7.42] | **⨁⨁**OO |
| C+QG vs C+TC | 1.44 [-6.99, 9.87] | **⨁⨁⨁**O | - | - | 1.44 [-6.99, 9.87] | **⨁⨁⨁**O |
| C+QG vs C+WQX | - | - | ‘-2.11 [-4.33, -0.11] | **⨁⨁**OO | ‘-2.11 [-4.33, -0.11] | **⨁⨁**OO |
| C+TC vs C | -3.71 [-6.49, -0.94] | **⨁⨁**OO | - | - | -3.71 [-6.49, -0.94] | **⨁⨁**OO |
| C+TC vs C+WQX | -0.2 [-2.22, 1.82] | **⨁⨁**OO | - | - | -0.2 [-2.22, 1.82] | **⨁⨁⨁**O |
| C+WQX vs C | -10.71 [-13.9, -7.53] | **⨁⨁**OO | - | - | -10.71 [-13.9, -7.53] | **⨁⨁**OO |

**QOL**

| **Comparison** | **Direct evidence** | | **Indirect evidence** | | **Network Meta-Analysis** | |
| --- | --- | --- | --- | --- | --- | --- |
|  | **MD[95%CI]** | **Certainty of evidence** | **MD[95%CI]** | **Certainty of evidence** | **MD[95%CI]** | **Certainty of evidence** |
| C+BDJ vs C | 6.87[3.69, 10.05] | **⨁⨁⨁**O | - | - | 6.87[3.69, 10.05] | **⨁⨁⨁**O |
| C+TC vs C | 9.04 [-4.8, 22.88] | **⨁⨁**OO | - | - | 9.04 [-4.8, 22.88] | **⨁⨁**OO |
| C+BDJ vs C+TC | - | - | ‘-2.17 [8.49, -12.83] | **⨁⨁**OO | ‘-2.17 [8.49, -12.83] | **⨁⨁**OO |

**PSQI**

| **Comparison** | **Direct evidence** | | **Indirect evidence** | | **Network Meta-Analysis** | |
| --- | --- | --- | --- | --- | --- | --- |
|  | **MD[95%CI]** | **Certainty of evidence** | **MD[95%CI]** | **Certainty of evidence** | **MD[95%CI]** | **Certainty of evidence** |
| C+BDJ vs C | -2.35[-3.13, -1.56] | **⨁⨁⨁**O | - | - | -2.35[-3.13, -1.56] | **⨁⨁⨁**O |
| C+TC vs C | -1.99[-3.38, -0.6] | **⨁⨁⨁⨁** | - | - | -1.99[-3.38, -0.6] | **⨁⨁⨁⨁** |
| C+BDJ vs C+TC | - | - | -0.36[0.25, -0.96] | **⨁⨁⨁**O |  | **⨁⨁⨁**O |

**TC**

| **Comparison** | **Direct evidence** | | **Indirect evidence** | | **Network Meta-Analysis** | |
| --- | --- | --- | --- | --- | --- | --- |
|  | **MD[95%CI]** | **Certainty of evidence** | **MD[95%CI]** | **Certainty of evidence** | **MD[95%CI]** | **Certainty of evidence** |
| C+BDJ vs C | -0.95 [-1.26, -0.64] | **⨁⨁⨁⨁** | - | - | -0.95 [-1.26, -0.64] | **⨁⨁⨁⨁** |
| C+QG vs C | -1.09 [-1.67, -0.52] | **⨁**OOO | - | - | -1.09 [-1.67, -0.52] | **⨁**OOO |
| C+TC vs C | -0.45 [-0.61, -0.29] | **⨁⨁⨁⨁** | - | - | -0.45 [-0.61, -0.29] | **⨁⨁⨁⨁** |
| C+BDJ vs C+QG | - | - | 0.14 [0.41, -0.12] | **⨁**OOO | 0.14 [0.41, -0.12] | **⨁**OOO |
| C+BDJ vs C+TC | - | - | ‘-0.5 [-0.65, -0.35] | **⨁⨁⨁⨁** | ‘-0.5 [-0.65, -0.35] | **⨁⨁⨁⨁** |
| C+QG vs C+TC | - | - | ’-0.64 [-1.06, -0.23] | **⨁**OOO | ’-0.64 [-1.06, -0.23] | **⨁**OOO |

**TG**

| **Comparison** | **Direct evidence** | | **Indirect evidence** | | **Network Meta-Analysis** | |
| --- | --- | --- | --- | --- | --- | --- |
|  | **MD[95%CI]** | **Certainty of evidence** | **MD[95%CI]** | **Certainty of evidence** | **MD[95%CI]** | **Certainty of evidence** |
| C+BDJ vs C | -0.22 [-0.52, 0.08] | **⨁⨁⨁**O | - | - | -0.22 [-0.52, 0.08] | **⨁⨁⨁**O |
| C+QG vs C | -0.55 [-0.82, -0.28] | **⨁⨁⨁**O | - | - | -0.55 [-0.82, -0.28] | **⨁⨁⨁**O |
| C+TC vs C | -0.28 [-0.4, -0.16] | **⨁⨁⨁⨁** | - | - | -0.28 [-0.4, -0.16] | **⨁⨁⨁⨁** |
| C+BDJ vs C+QG | - | - | 0.33 [0.3, 1.08] | **⨁⨁⨁**O | 0.33 [0.3, 1.08] | **⨁⨁⨁**O |
| C+BDJ vs C+TC | - | - | 0.06 [-0.12, 0.24] | **⨁⨁⨁**O | 0.06 [-0.12, 0.24] | **⨁⨁⨁**O |
| C+QG vs C+TC | - | - | -0.27 [-0.42, -0.12] | **⨁⨁⨁**O | -0.27 [-0.42, -0.12] | **⨁⨁⨁**O |

**HDL-C**

| **Comparison** | **Direct evidence** | | **Indirect evidence** | | **Network Meta-Analysis** | |
| --- | --- | --- | --- | --- | --- | --- |
|  | **MD[95%CI]** | **Certainty of evidence** | **MD[95%CI]** | **Certainty of evidence** | **MD[95%CI]** | **Certainty of evidence** |
| C+BDJ vs C | 0.13 [-0.2, 0.46] | **⨁⨁⨁**O | - | - | 0.13 [-0.2, 0.46] | **⨁⨁⨁**O |
| C+QG vs C | 0.25 [0, 0.5] | **⨁⨁⨁**O | - | - | 0.25 [0, 0.5] | **⨁⨁⨁**O |
| C+TC vs C | 0.13 [-0.02, 0.28] | **⨁**OOO | - | - | 0.13 [-0.02, 0.28] | **⨁**OOO |
| C+BDJ vs C+QG | - | - | ‘-0.12 [-0.2, -0.06] | **⨁⨁⨁**O | ‘-0.12 [-0.2, -0.06] | **⨁⨁⨁**O |
| C+BDJ vs C+TC | - | - | 0 [-0.18, 0.18] | **⨁**OOO | 0 [-0.18, 0.18] | **⨁**OOO |
| C+QG vs C+TC | - | - | 0.12 [0.02, 0.22] | **⨁**OOO | 0.12 [0.02, 0.22] | **⨁**OOO |

**LDL-C**

| **Comparison** | **Direct evidence** | | **Indirect evidence** | | **Network Meta-Analysis** | |
| --- | --- | --- | --- | --- | --- | --- |
|  | **MD[95%CI]** | **Certainty of evidence** | **MD[95%CI]** | **Certainty of evidence** | **MD[95%CI]** | **Certainty of evidence** |
| C+QG vs C | -1.1[-1.62, -0.58] | **⨁**OOO | - | - | -1.1[-1.62, -0.58] | **⨁**OOO |
| C+TC vs C | -0.32[-0.44, -0.2] | **⨁⨁⨁⨁** | - | - | -0.32[-0.44, -0.2] | **⨁⨁⨁⨁** |
| C+QG vs C+TC | - | - | 0.21[-1.18, -0.38] | **⨁**OOO | 0.21[-1.18, -0.38] | **⨁**OOO |

**HR**

| **Comparison** | **Direct evidence** | | **Indirect evidence** | | **Network Meta-Analysis** | |
| --- | --- | --- | --- | --- | --- | --- |
|  | **MD[95%CI]** | **Certainty of evidence** | **MD[95%CI]** | **Certainty of evidence** | **MD[95%CI]** | **Certainty of evidence** |
| C+BDJ vs C | -4.39 [-5.05, -3.74] | **⨁⨁⨁⨁** | - | - | -4.39 [-5.05, -3.74] | **⨁⨁⨁⨁** |
| C+TC vs C | -2.73 [-6.31, 0.85] | **⨁⨁**OO | - | - | -2.73 [-6.31, 0.85] | **⨁⨁**OO |
| C+WQX vs C | -3.51 [-8.44, 1.41] | **⨁**OOO | - | - | -3.51 [-8.44, 1.41] | **⨁**OOO |
| C+TC vs C+WQX | 3.36 [-0.39, 7.11] | **⨁⨁⨁**O | - | - | 3.36 [-0.39, 7.11] | **⨁⨁⨁**O |
| C+BDJ vs C+TC | - | - | -1.66 [1.26, -4.59] | **⨁⨁**OO | -1.66 [1.26, -4.59] | **⨁⨁**OO |
| C+BDJ vs C+WQX | - | - | -0.88 [3.39, -5.15] | **⨁**OOO | -0.88 [3.39, -5.15] | **⨁**OOO |

**Note: ⨁**OOO: Very low; **⨁⨁**OO: Low; **⨁⨁⨁**O: Moderate; **⨁⨁⨁⨁**: High

**Table S3.** Inconsistency of SBP, DBP, and HR tested by loop-specific heterogeneity estimates, inconsistency model and node splitting analysis.

| **loop-specific heterogeneity estimates** | | | | | | | | | |
| --- | --- | --- | --- | --- | --- | --- | --- | --- | --- |
| **Loop** | **IF** | | **seIF** | **z_value** | **p_value** | | **CI_95** | | **Loop_Hete rog_tau2** |
| **SBP** | | | | | | | | | |
| C-(C+TC)-(C+WQX) | 5.946 | | 10.016 | 0.594 | 0.553 | | (0.00,25.58) | | 63.388 |
| C-(C+QG)-(C+TC) | 5.847 | | 14.322 | 0.408 | 0.683 | | (0.00,33.92) | | 75.745 |
| **DBP** | | | | | | | | | |
| C-(C+QG)-(C+TC) | 7.653 | | 8.802 | 0.870 | 0.385 | | (0.00,24.90) | | 23.836 |
| C-(C+TC)-(C+WQX) | 4.022 | | 1.846 | 2.179 | 0.029 | | (0.00,22.05) | | 23.316 |
| **HR** | | | | | | | | | |
| C-(C+TC)-(C+WQX) | | 5.030 | 2.916 | 1.725 | | 0.085 | | (0.00,10.74) | 0.000 |

**Note:** A-C, B-C+BDJ, C-C+QG, D-C+TC, E-C+WQX

| **Inconsistency model** | | | |
| --- | --- | --- | --- |
|  | **SBP** | **DBP** | **HR** |
| chi2 | 5.33 | 2.44 | 6.02 |
| Prob > chi2 | 0.3767 | 0.4854 | 0.0141 |

| **Node splitting analysis** | | | | | | | | |
| --- | --- | --- | --- | --- | --- | --- | --- | --- |
| **Side** | | **Direct** |  | **Indirect** |  | **Difference** |  |  |
|  | | **Coef.** | **Std. Err.** | **Coef.** | **Std. Err.** | **Coef.** | **Std. Err.** | **P>z** |
| SBP | | | | | | | | |
| A B | | . | . | . | . | . | . | . |
| A C * | | -16.4596 | 5.014621 | -11.46508 | 16.74925 | -4.994515 | 17.50872 | 0.775 |
| A D | | -8.701938 | 2.133987 | -12.02268 | 8.909826 | 3.320746 | 9.16152 | 0.717 |
| A E | | -18.14966 | 4.69002 | -7.944858 | 7.464636 | -10.2048 | 8.805397 | 0.246 |
| C D | | 1.69426 | 9.278161 | 9.486014 | 6.035052 | -7.791754 | 11.07465 | 0.482 |
| D E | | -4.407621 | 5.78251 | -8.499844 | 5.978171 | 4.092223 | 8.313363 | 0.623 |
| DBP | | | | | | | | |
| A B | | . | . | . | . | . | . | . |
| A C * | | -12.56879 | 3.517128 | -2.78169 | 11.50109 | -9.787101 | 11.87821 | 0.41 |
| A D | | -3.722805 | 1.466729 | -12.39043 | 5.941137 | 8.667626 | 6.117811 | 0.157 |
| A E | | -10.97077 | 3.998014 | -3.676163 | 5.450563 | -7.294609 | 6.759551 | 0.281 |
| C D | | 1.53944 | 6.691057 | 9.954382 | 4.194311 | -8.414942 | 7.821126 | 0.282 |
| D E | | 0.1999997 | 5.247557 | -7.094549 | 4.260844 | 7.294548 | 6.75956 | 0.281 |
| HR | | | | | | | | |
| A B | . | | . | . | . | . | . | . |
| A C * | -2.73 | | 1.905599 | 7.33 | 4.034576 | -10.06 | 4.099012 | 0.014 |
| A D | . | | . | . | . | . | . | . |
| C D * | -3.36 | | 1.99052 | 6.7 | 3.909632 | -10.06 | 4.099011 | 0.014 |

* All the evidence about these contrasts comes from the trials which directly compare them.

**Table S4.** Ranking of exercise interventions in order of effectiveness.

| SBP | |  | DBP | |  | Total cholesterol | |
| --- | --- | --- | --- | --- | --- | --- | --- |
| Treatment | SUCRA（%） |  | Treatment | SUCRA（%） |  | Treatment | SUCRA（%） |
| C+WQX | 85.7 |  | C+QG | 76.826 |  | C+BDJ | 95.1 |
| C+QG | 74.4 |  | C+WQX | 76.824 |  | C+QG | 66.3 |
| C+BDJ | 44.9 |  | C+BDJ | 60.2 |  | C+TC | 38.1 |
| C+TC | 44.7 |  | C+TC | 35.2 |  | C | 0.5 |
| C | 0.3 |  | C | 0.09 |  |  |  |
| HDL-C | |  | LDL-C | |  | TG | |
| Treatment | SUCRA（%） |  | Treatment | SUCRA（%） |  | Treatment | SUCRA（%） |
| C+QG | 64.5 |  | C+BDJ | 95.1 |  | C+BDJ | 95.1 |
| C+BDJ | 63.7 |  | C+QG | 66.3 |  | C+QG | 66.3 |
| C+TC | 55.9 |  | C+TC | 38.1 |  | C+TC | 38.1 |
| C | 15.9 |  | C | 0.5 |  | C | 0.5 |
| HR | |  | QOL | |  | PSQI | |
| Treatment | SUCRA（%） |  | Treatment | SUCRA（%） |  | Treatment | SUCRA（%） |
| C+BDJ | 83.4 |  | C+BDJ | 72.5 |  | C+BDJ | 80.5 |
| C+WQX | 65.1 |  | C+TC | 78.6 |  | C+TC | 64.1 |
| C+TC | 37.6 |  | C | 8.9 |  | C | 5.4 |
| C | 13.9 |  |  |  |  |  |  |

**Robustness of network meta-analysis results after excluding studies with moderate-to-high risk of bias: SUCRA-based ranking based on low-risk studies.**

| SBP | |  | DBP | |  | Total cholesterol | |
| --- | --- | --- | --- | --- | --- | --- | --- |
| Treatment | SUCRA（%） |  | Treatment | SUCRA（%） |  | Treatment | SUCRA（%） |
| C+WQX | 85.6 |  | C+WQX | 85.6 |  | C+BDJ | 96.6 |
| C+QG | 74.3 |  | C+QG | 74.3 |  | C+TC | 52.8 |
| C+BDJ | 44.0 |  | C+BDJ | 45.7 |  | C | 0.5 |
| C+TC | 44.7 |  | C+TC | 44.0 |  |  |  |
| C | 0.4 |  | C | 0.09 |  |  |  |
| HDL-C | |  | LDL-C | |  | TG | |
| Treatment | SUCRA（%） |  | Treatment | SUCRA（%） |  | Treatment | SUCRA（%） |
| C+BDJ | 70.40 |  | C+TC | 99.8 |  | C+BDJ | 81.3 |
| C+TC | 58.2 |  | C | 0.1 |  | C+TC | 58.6 |
| C | 21.4 |  |  |  |  | C | 0.5 |
|  |  |  |  |  |  |  |  |
| HR | |  | QOL | |  | PSQI | |
| Treatment | SUCRA（%） |  | Treatment | SUCRA（%） |  | Treatment | SUCRA（%） |
| C+BDJ | 93.5 |  | C+BDJ | 72.5 |  | C+BDJ | 80.5 |
| C+WQX | 39.4 |  | C+TC | 78.6 |  | C+TC | 64.1 |
| C | 17.0 |  | C | 8.9 |  | C | 5.4 |
|  |  |  |  |  |  |  |  |

**Table S5.** Eegg's test for the primary and secondary outcomes.

| SBP | |  | DBP | |  | Total cholesterol | |
| --- | --- | --- | --- | --- | --- | --- | --- |
| t | p |  | t | p |  | t | p |
| 3.67 | 0.0007 |  | 0.46 | 0.6471 |  | 0.61 | 0.5560 |
| HDL-C | |  | LDL-C (k=9) | |  | TG | |
| t | p |  | t | p |  | t | p |
| -1.83 | 0.1048 |  | - | - |  | 0.41 | 0.6931 |
| HR(k=6) | |  | QOL (k=7) | |  | PSQI (k=5) | |
| t | p |  | t | p |  | t | p |
| - | - |  | - | - |  | - | - |

Table S6 Characteristics of the included studies

| Author | Diagnostic criteria for hypertension | The diagnostic cut-off value of hypertension | Details of intervention in the control group | Details of exercise in the treatment group |
| --- | --- | --- | --- | --- |
| Wang XB et al | Chinese Guidelines for the prevention and treatment of hypertension 2010 | SBP ≥140 mmHg and/or DBP ≥90 mmHg | Conventional drugs | Tai Chi, 3mos, three times a week for 40-60 minutes |
| Liu T et al | Chinese Guidelines for the prevention and treatment of hypertension 2010 | SBP ≥140 mmHg and/or DBP ≥90 mmHg | Cilazapril (2.5 mg /d) | Tai Chi, 6mos, four to six sessions per week for 60 minutes each |
| Chen DZ | Chinese Guidelines for the prevention and treatment of hypertension 2010 | SBP ≥140 mmHg and/or DBP ≥90 mmHg | Received no intervention | Qigong,6mos, five sessions per week for 60 minutes |
| Xiao CM et al | Chinese Guidelines for the prevention and treatment of hypertension 2010 | SBP ≥140 mmHg and/or DBP ≥90 mmHg | Received no intervention | Baduanjin, 6mos, five sessions per week for 60 minutes |
| Feng LJ et al | Chinese Guidelines for the prevention and treatment of hypertension 2016 | SBP ≥140 mmHg and/or DBP ≥90 mmHg | Education about hypertension, medication use | Tai Chi, 6mos, five sessions per week for 60 minutes |
| Ma CH et al | Chinese Guidelines for the prevention and treatment of hypertension 2016 | SBP ≥140 mmHg and/or DBP ≥90 mmHg | Medication use | Tai Chi, 12W, three sessions per week were given for 60 minutes |
| Hedo RY | Chinese Guidelines for the prevention and treatment of hypertension 2018 | SBP ≥140 mmHg and/or DBP ≥90 mmHg | Thiazide diuretic, Gastrodia-gouteng drink | Tai Chi, 12W, three sessions per week were given for 60 minutes |
| Zhang DL | Chinese Guidelines for the prevention and treatment of hypertension 2010 | SBP ≥140 mmHg and/or DBP ≥90 mmHg | Amlodipine besylate tablets (5mg /day) | Tai Chi, 12W, three sessions per week were given for 60 minutes |
| Pan HS et al | Chinese Guidelines for the prevention and treatment of hypertension 2005 | SBP ≥140 mmHg and/or DBP ≥90 mmHg | Amlodipine (5mg /day) | Baduanjin, 24W, five sessions per week for 45 minutes |
| Lin F et al | Chinese Guidelines for the prevention and treatment of hypertension 2010 | SBP ≥140 mmHg and/or DBP ≥90 mmHg | Control blood pressure, blood glucose and blood lipids | Baduanjin, 12W. The sessions were conducted 14 times per week for 30 minutes |
| Liang HY et al | Chinese Guidelines for the prevention and treatment of hypertension 2010 | SBP ≥140 mmHg and/or DBP ≥90 mmHg | Amlodipine was given once a day, 5 mg each time. Deanxit 0.5-1 tablets /d | Baduanjin,3mos,five sessions per week for 45 minutes |
| Luo F | Chinese Guidelines for the prevention and treatment of hypertension 2010 | SBP ≥140 mmHg and/or DBP ≥90 mmHg | Medication care, diet management, blood pressure monitoring and psychological counseling | Baduanjin, 12W. The sessions were conducted 3 times a week for 40 minutes |
| Jiang YH | Chinese Guidelines for the prevention and treatment of hypertension 2010 | SBP ≥140 mmHg and/or DBP ≥90 mmHg | Dihydropyridine calcium channel blockers | Baduanjin, 12W, The sessions were conducted 14 times per week for 30 minutes |
| Zheng LW et al-a | Chinese Guidelines for the prevention and treatment of hypertension 2019 | SBP ≥140 mmHg and/or DBP ≥90 mmHg | Take hypertension medications correctly | Baduanjin, 12W, The sessions were performed 10 times a week for 13 minutes |
| Zheng LW et al-b | Chinese Guidelines for the prevention and treatment of hypertension 2018 | SBP ≥140 mmHg and/or DBP ≥90 mmHg | Take hypertension medications correctly | Baduanjin, 12W. The sessions were performed 10 times a week for 13 minutes |
| Yang WW et al | Chinese Guidelines for the prevention and treatment of hypertension 2018 | SBP ≥140 mmHg and/or DBP ≥90 mmHg | Antihypertensive drugs | Baduanjin, 12W. The sessions were conducted 10 times a week for 30 minutes |
| Tan HL et al | Chinese Guidelines for the prevention and treatment of hypertension 2018 | SBP ≥140 mmHg and/or DBP ≥90 mmHg | Antihypertensive drugs | Baduanjin, 12W. The sessions were conducted seven times a week for 30 minutes |
| Fan WY et al | Chinese Guidelines for the prevention and treatment of hypertension 2018 | SBP ≥140 mmHg and/or DBP ≥90 mmHg | Valsartan capsule, estazolam tablet | Baduanjin, 12W, The sessions were performed 10 times a week for 13 minutes |
| Zheng LW et al | Chinese Guidelines for the prevention and treatment of hypertension 2005 | SBP ≥140 mmHg and/or DBP ≥90 mmHg | Lecardipine tablets, health education | Baduanjin, 12W, five times a week for 30 minutes each session |
| Chen LH | Chinese Guidelines for the prevention and treatment of hypertension 2010 | SBP ≥140 mmHg and/or DBP ≥90 mmHg | Ambroxol or roxithromycin | Baduanjin, 12W, five sessions per week for 60 minutes |
| Tang QH | 1999 WHO | SBP ≥140 mmHg and/or DBP ≥90 mmHg | Antihypertensive drugs | Tai Chi, 6mos, five sessions per week for 60 minutes |
| Yi R et al | 1995 WHO | SBP ≥140 mmHg and/or DBP ≥90 mmHg | Cilazapril (25mg/d) | Tai Chi, 20W, five sessions per week for 60 minutes |
| Zhang XY et al | Chinese Guidelines for the prevention and treatment of hypertension 2018 | SBP ≥140 mmHg and/or DBP ≥90 mmHg | Health nursing education, antihypertensive drugs | Baduanjin, 24W, three sessions per week for 60 minutes |
| Xu DM | 1999 WHO | SBP ≥140 mmHg and/or DBP ≥90 mmHg | Health nursing education, antihypertensive drugs | Tai Chi, 20W, five sessions per week for 60 minutes |
| Hu LX et al | Chinese Guidelines for the prevention and treatment of hypertension 2010 | SBP ≥140 mmHg and/or DBP ≥90 mmHg | Health nursing education, antihypertensive drugs | Tai Chi, 3M, five sessions per week for 45 minutes |
| Kong XZ et al | Chinese Guidelines for the prevention and treatment of hypertension 2018 | SBP ≥140 mmHg and/or DBP ≥90 mmHg | Drug therapy | Baduanjin, 2W. The sessions were conducted 14 times per week for 30 minutes |
| Lin Qiu et al | Chinese Guidelines for the prevention and treatment of hypertension 2010 | SBP ≥140 mmHg and/or DBP ≥90 mmHg | Amlodipine (5mg/day) | Baduanjin, 6M. The sessions were conducted 14 times per week for 30 minutes |
| Lin Hong et al | 1999 WHO | SBP ≥140 mmHg and/or DBP ≥90 mmHg | Hypotensor | Wuqinxi, 6M, six sessions per week for 60 minutes |
| He X | Chinese Guidelines for the prevention and treatment of hypertension 2010 | SBP ≥140 mmHg and/or DBP ≥90 mmHg | Conventional drugs | Baduanjin, 3M, five sessions per week for 30 minutes |
| Hu JC | Chinese Guidelines for the prevention and treatment of hypertension 2010 | SBP ≥140 mmHg and/or DBP ≥90 mmHg | Do not participate in any physical exercise | Tai Chi, 12W, seven sessions per week for 60 minutes |
| Xie HJ et al | Chinese Guidelines for the prevention and treatment of hypertension 2010 | SBP ≥140 mmHg and/or DBP ≥90 mmHg | Hypotensive drugs | Tai Chi, 12W, five sessions per week for 60 minutes |
| Cao YM et al | Chinese Guidelines for the prevention and treatment of hypertension 2010 | SBP ≥140 mmHg and/or DBP ≥90 mmHg | Levamlodipine maleate tablets (2.5mg/day) | Tai Chi, 3M, five sessions per week for 60 minutes |
| Sun F et al | Chinese Guidelines for the prevention and treatment of hypertension 2010 | SBP ≥140 mmHg and/or DBP ≥90 mmHg | Health education | Tai Chi, 8W, seven sessions per week for 120 minutes |
| Wei YH et al | Chinese Guidelines for the prevention and treatment of hypertension 2010 | SBP ≥140 mmHg and/or DBP ≥90 mmHg | Hypotensive drugs, health education, | Tai Chi, 6M, seven sessions per week for 45 minutes |
| Li W et al | Chinese Guidelines for the prevention and treatment of hypertension 2010 | SBP ≥140 mmHg and/or DBP ≥90 mmHg | Hypotensive drugs, health education, | Wuqinxi, 6M, six sessions per week for 30 minutes |
| Shen AM et al | Chinese Guidelines for the prevention and treatment of hypertension 2010 | SBP ≥140 mmHg and/or DBP ≥90 mmHg | Hypotensive drugs | Wuqinxi, 6M, six sessions per week for 60 minutes |
| Li CY et al | Chinese Guidelines for the prevention and treatment of hypertension 2010 | SBP ≥140 mmHg and/or DBP ≥90 mmHg | Captopril antihypertensive tablets | Tai Chi, 2M. The sessions were conducted 14 times per week for 40 minutes |
| Lian YL et al | Chinese Guidelines for the prevention and treatment of hypertension 2010 | SBP ≥140 mmHg and/or DBP ≥90 mmHg | Hypotensive drugs, health education, | Baduanjin, 6M, five sessions per week for 30 minutes |
| Chen WW et al | Chinese Guidelines for the prevention and treatment of hypertension 2015 | SBP ≥140 mmHg and/or DBP ≥90 mmHg | Hypotensive drugs | Baduanjin, 6M, five sessions per week for 30 minutes |
| Wu GY et al | Chinese Guidelines for the prevention and treatment of hypertension 2024 | SBP ≥140 mmHg and/or DBP ≥90 mmHg | Hypotensive drugs | Tai Chi, 6M. The sessions were conducted 14 times per week for 30 minutes |
| Lian YL et al | Chinese Guidelines for the prevention and treatment of hypertension 2018 | SBP ≥140 mmHg and/or DBP ≥90 mmHg | Hypotensive drugs, health education, | Baduanjin, 6M, five sessions per week for 30 minutes |
| Yao KR | Chinese Guidelines for the prevention and treatment of hypertension 2018 | SBP ≥140 mmHg and/or DBP ≥90 mmHg | Hypotensive drugs | Baduanjin, 3M, five sessions per week for 30 minutes |
| Wang P | Chinese Guidelines for the prevention and treatment of hypertension 2018 | SBP ≥140 mmHg and/or DBP ≥90 mmHg | Hypotensive drugs, health education, | Tai Chi, 3M, three sessions per week for 60 minutes |
| Yang XW | Chinese Guidelines for the prevention and treatment of hypertension 2010 | SBP ≥140 mmHg and/or DBP ≥90 mmHg | Hypotensive drugs | Tai Chi, 16W, three sessions per week for 60 minutes |

**
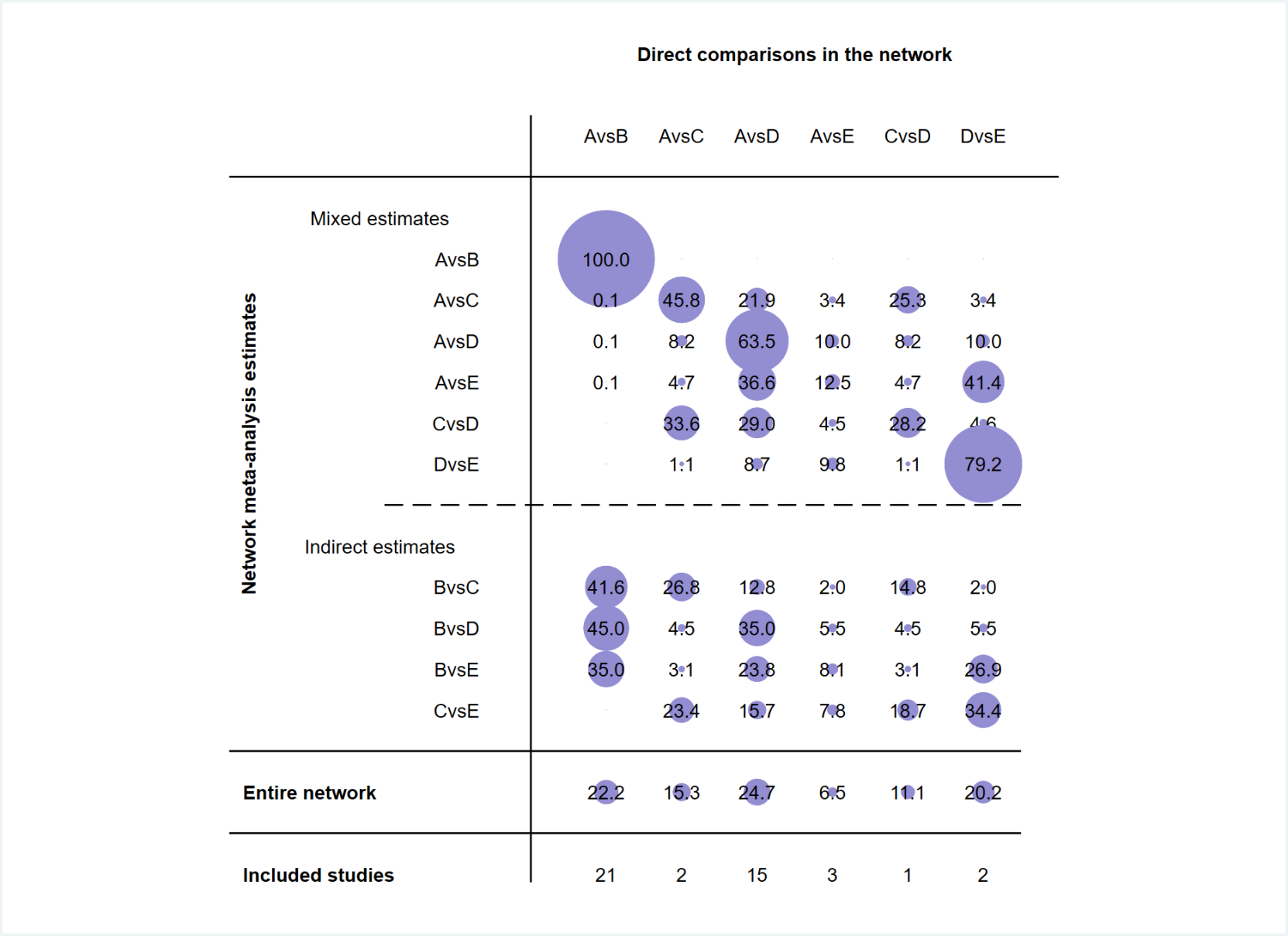
**

**Figure S1A** SBP.


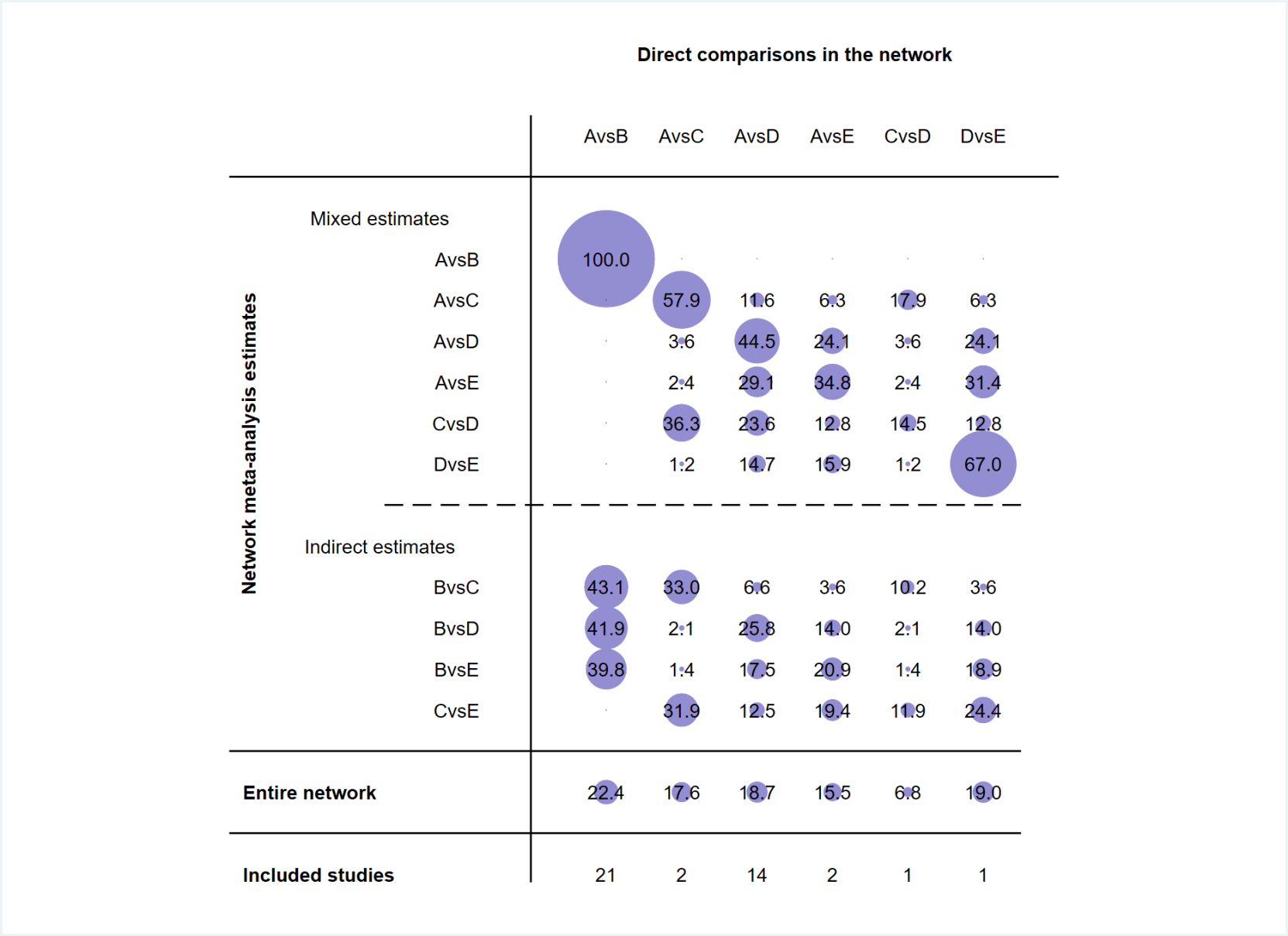


**Figure S1B** DBP.


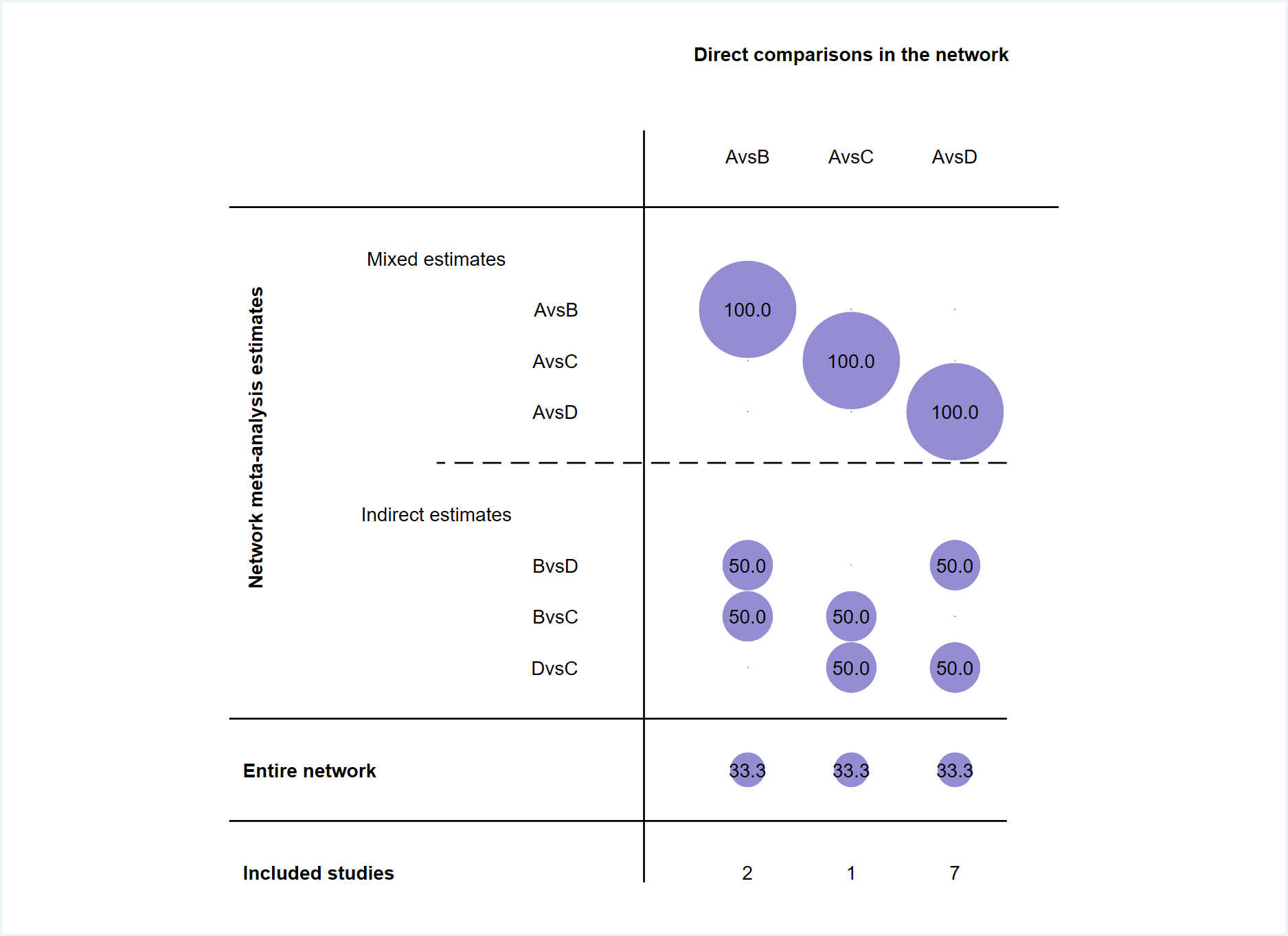


**Figure S1C** TC.


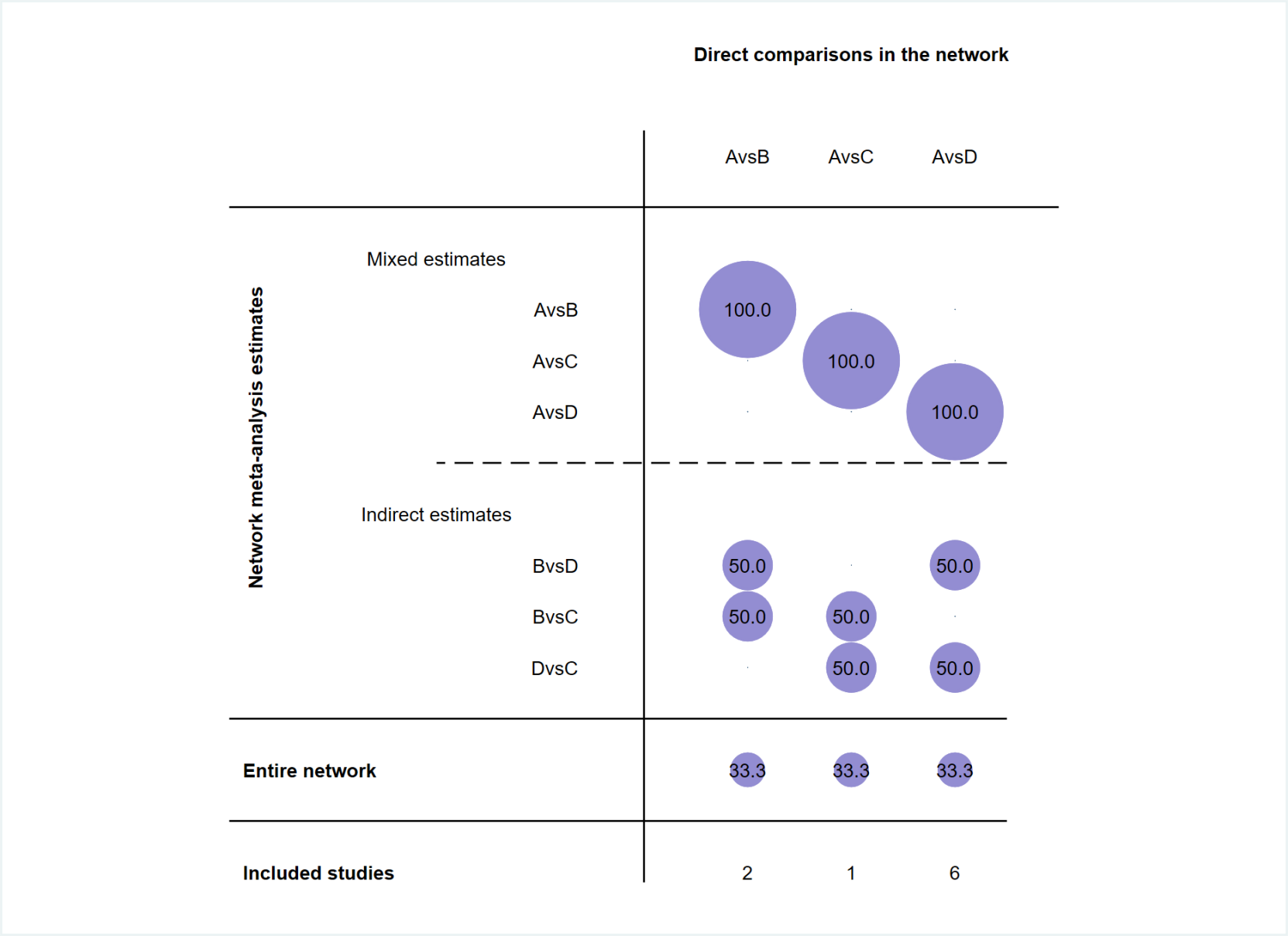


**Figure S1D** TG.


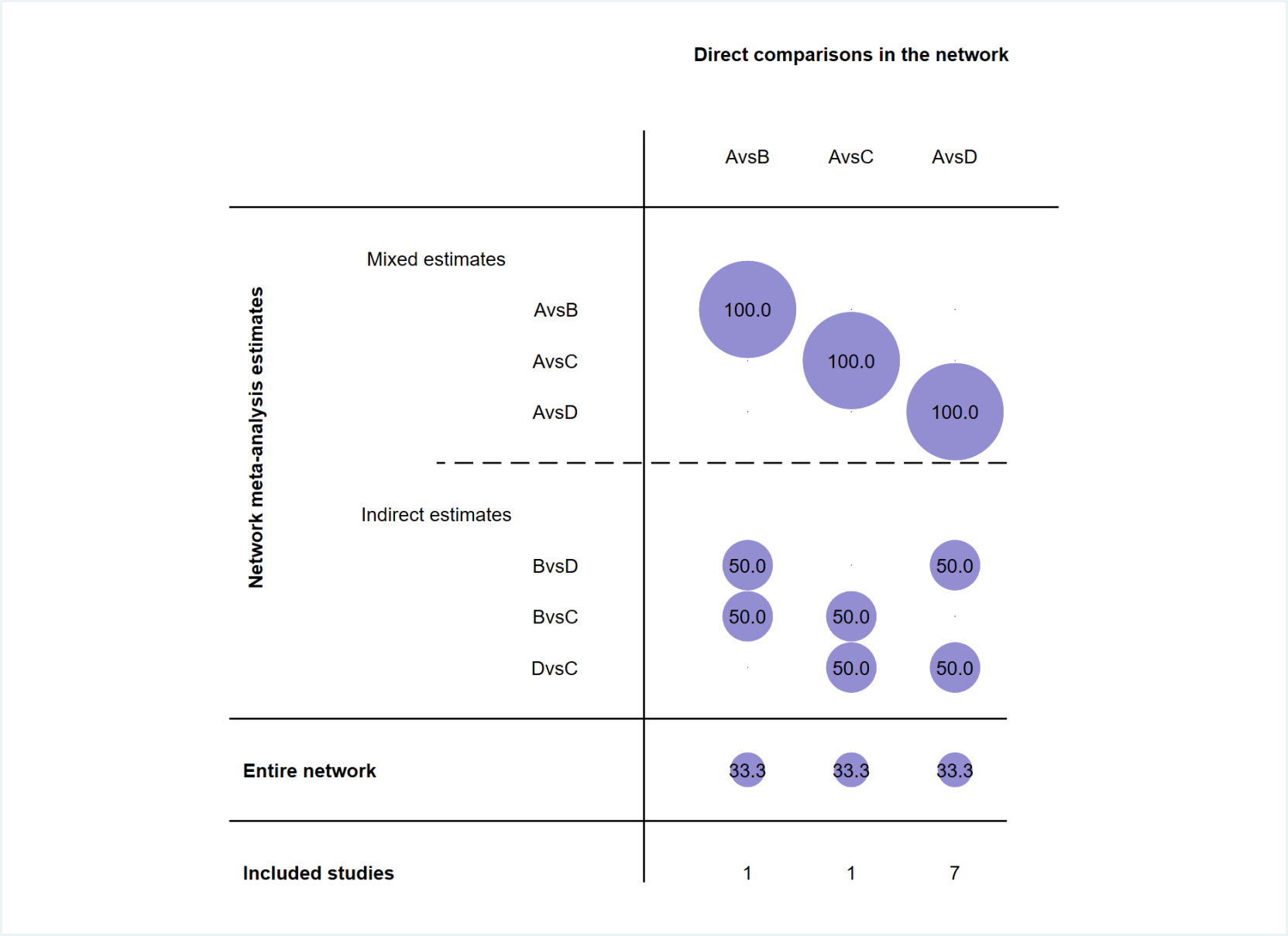


**Figure S1E** LDL-C.


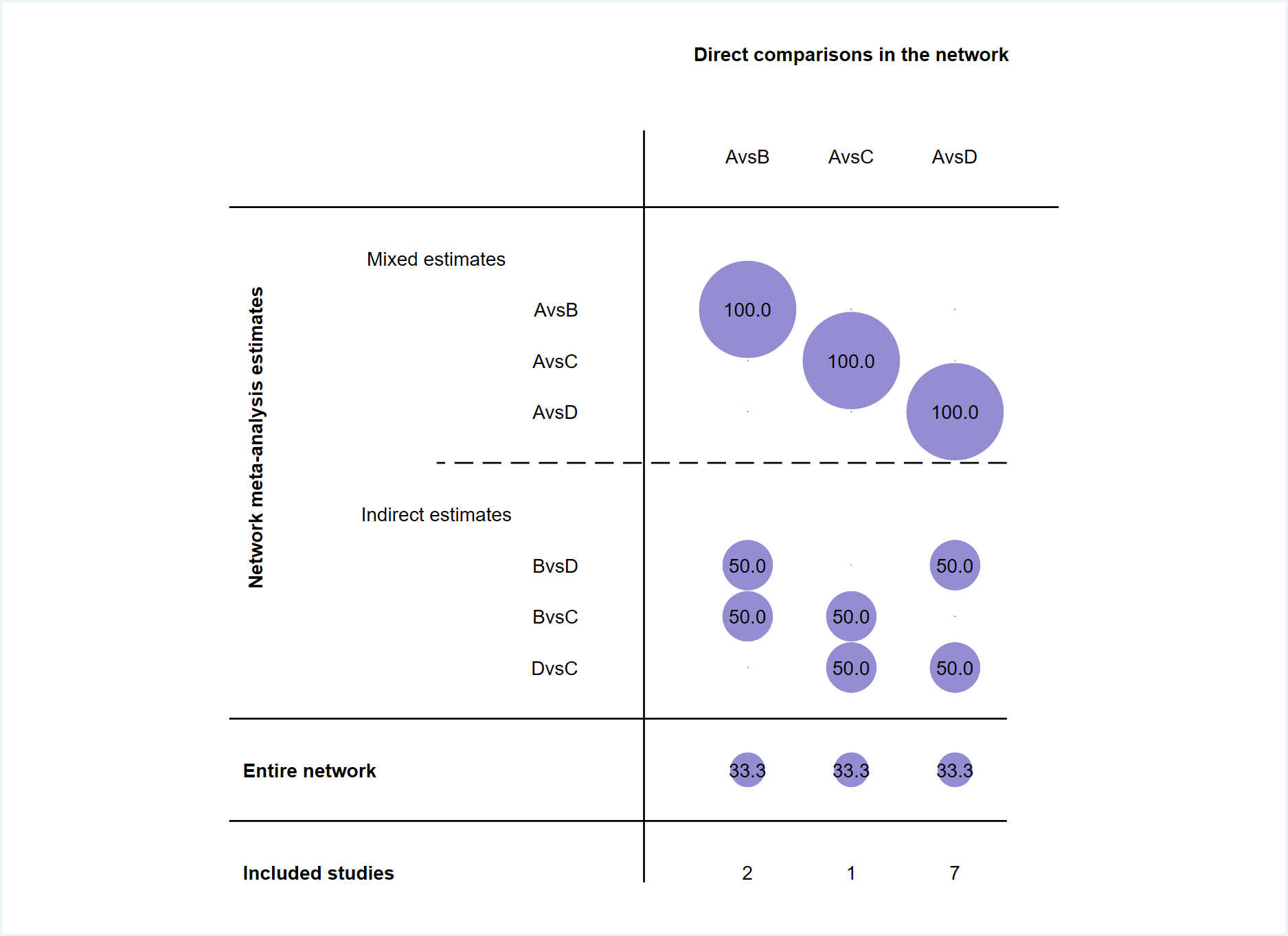


**Figure S1F** HDL-C.

**
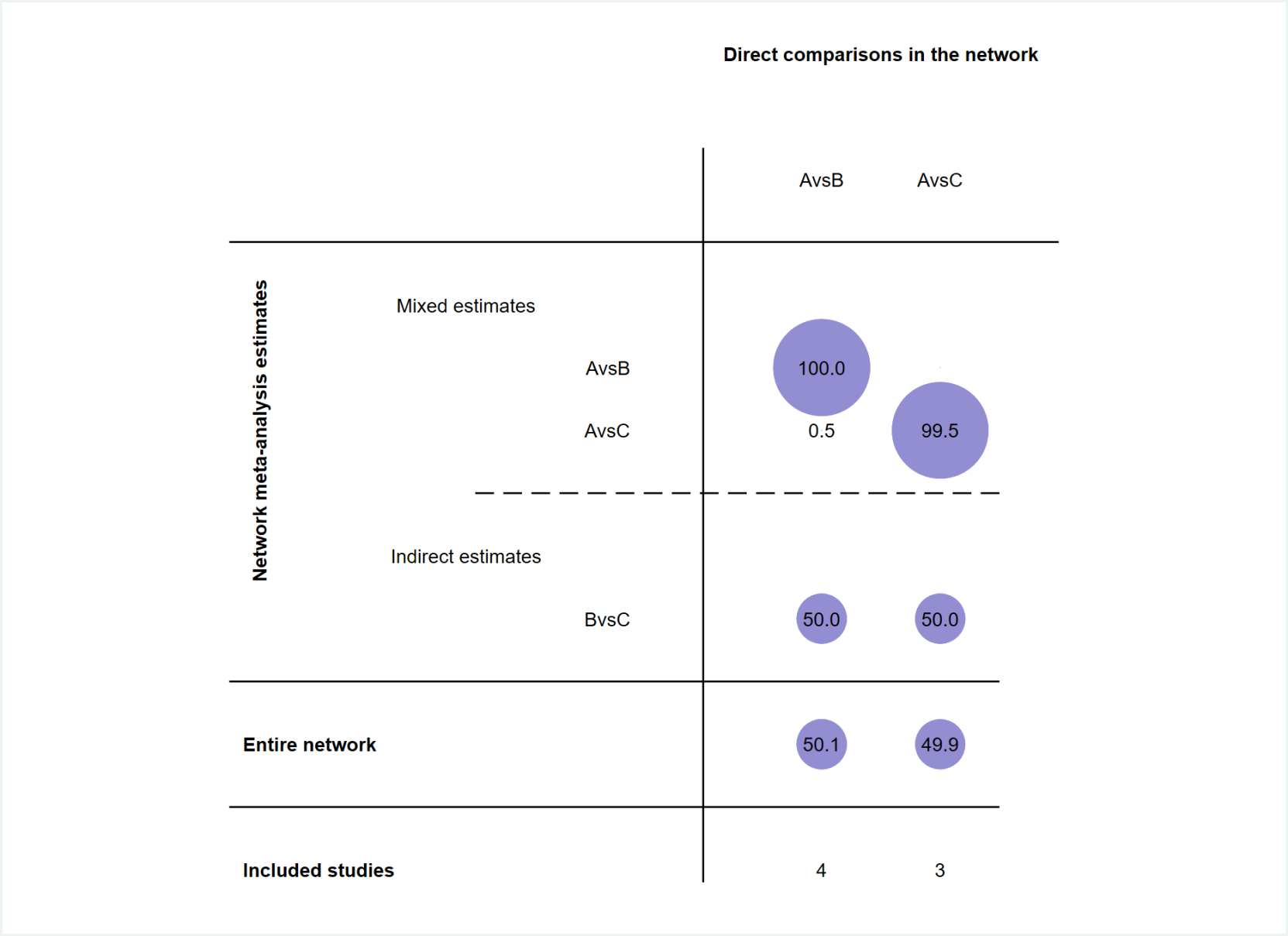
**

**Figure S1G** QOL.

**
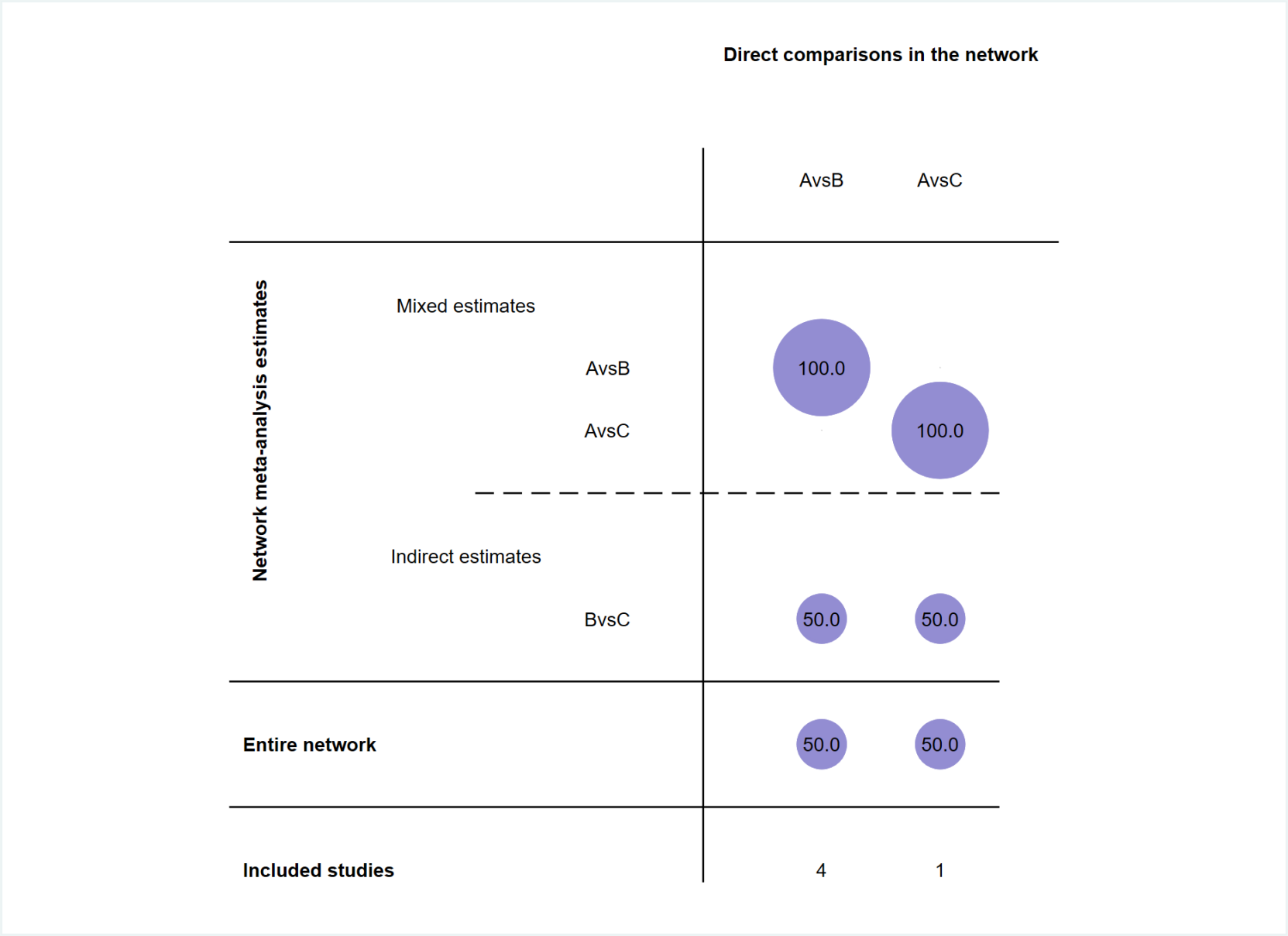
**

**Figure S1H** PSQI.

**
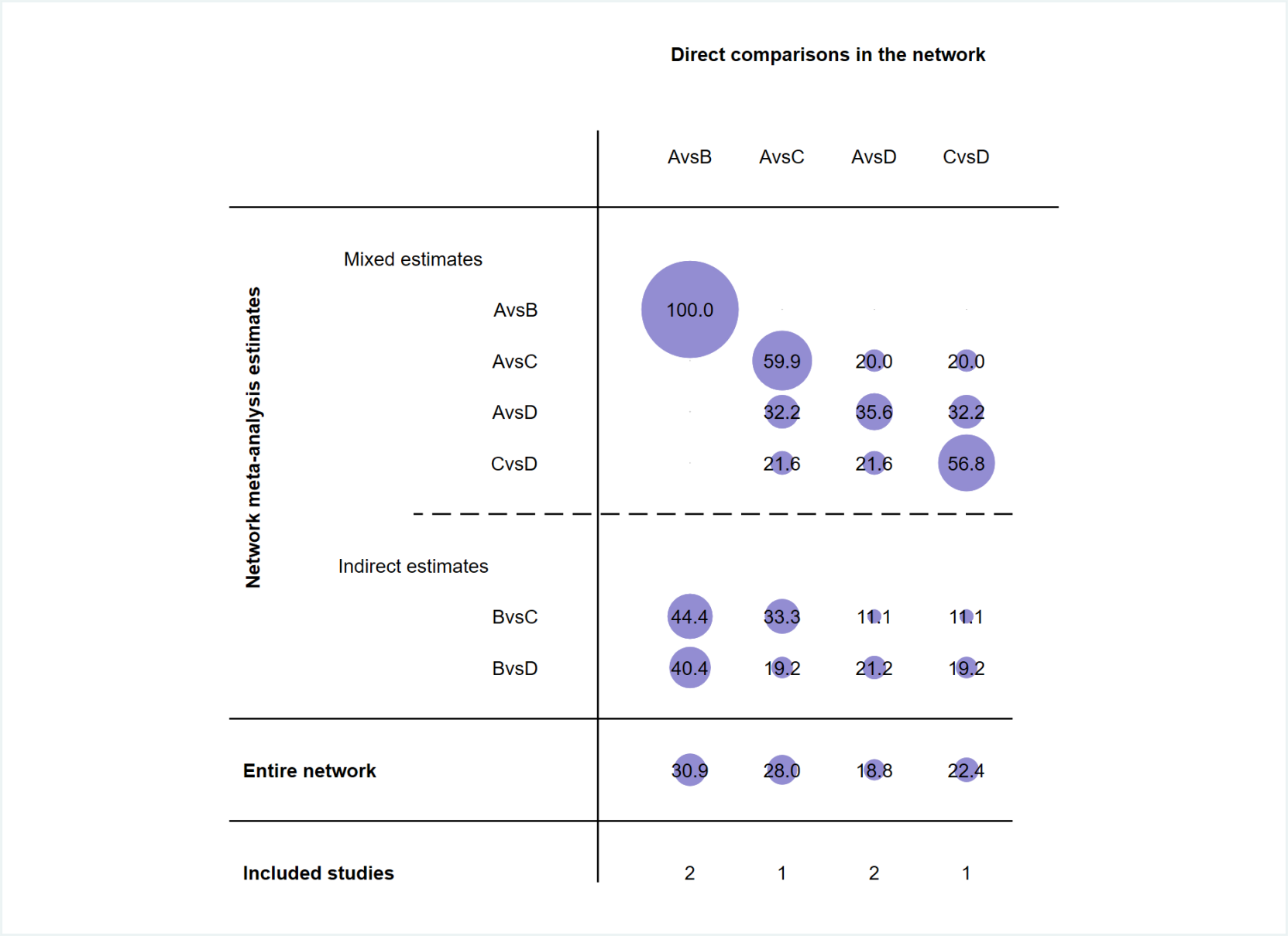
**

**Figure S1I HR**.

**Figure S1.** Contributions of direct and indirect comparisons to NMA and the number of studies of each direct comparison.

**A.** Systolic Blood Pressure; **B.** Diastolic Blood Pressure; **C.** Total cholesterol; **D.** Triglyceride; **E.** Low-density lipoprotein cholesterol; **F.** High-density lipoproteincholesterol; **G.** Quality of life; **H.** Pittsburgh sleep quality index; **I.** Heart rate

**Note:** A-C, B-C+BDJ, C-C+QG, D-C+TC, E-C+WQX


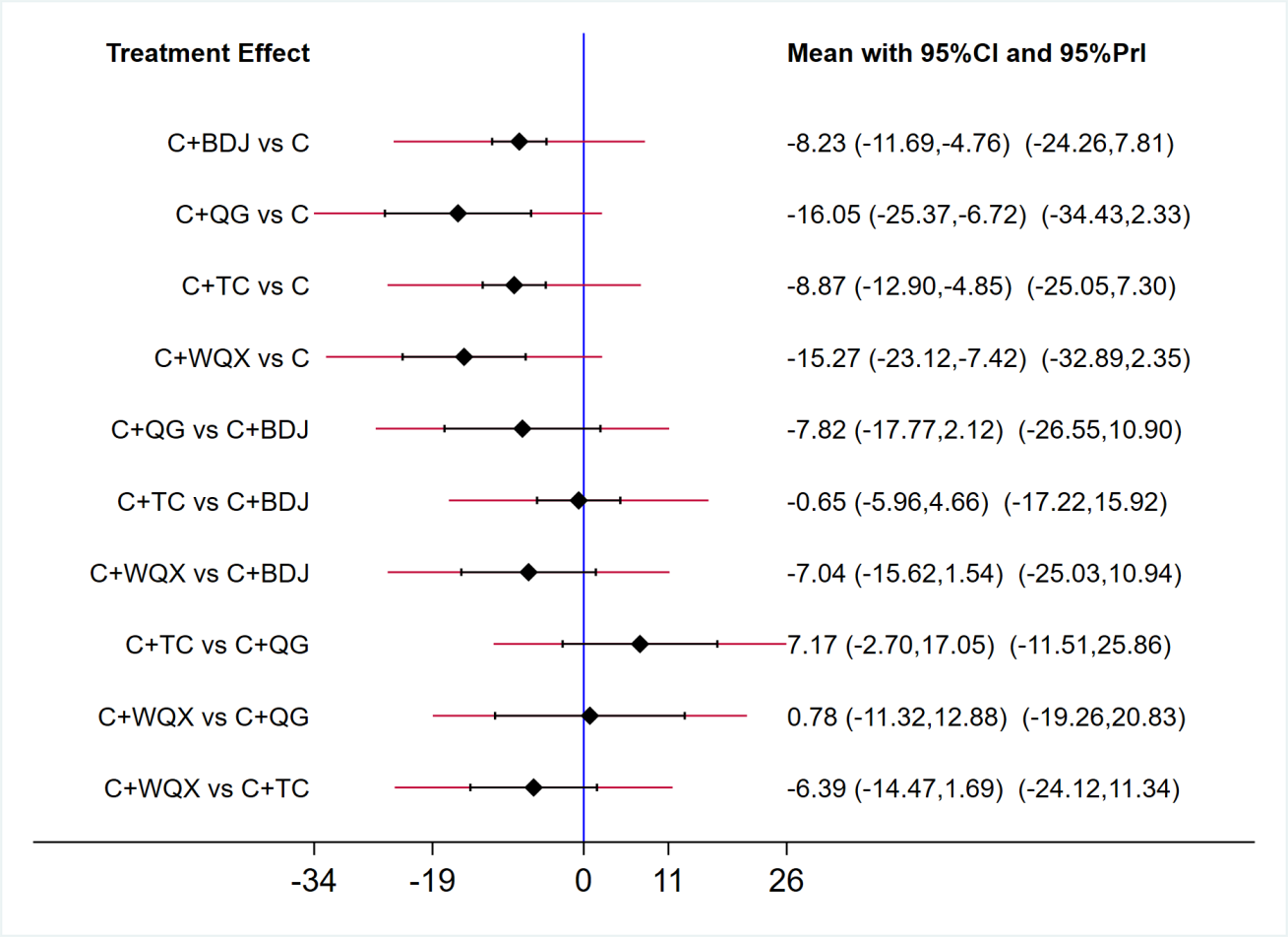


**Figure S2A** SBP.


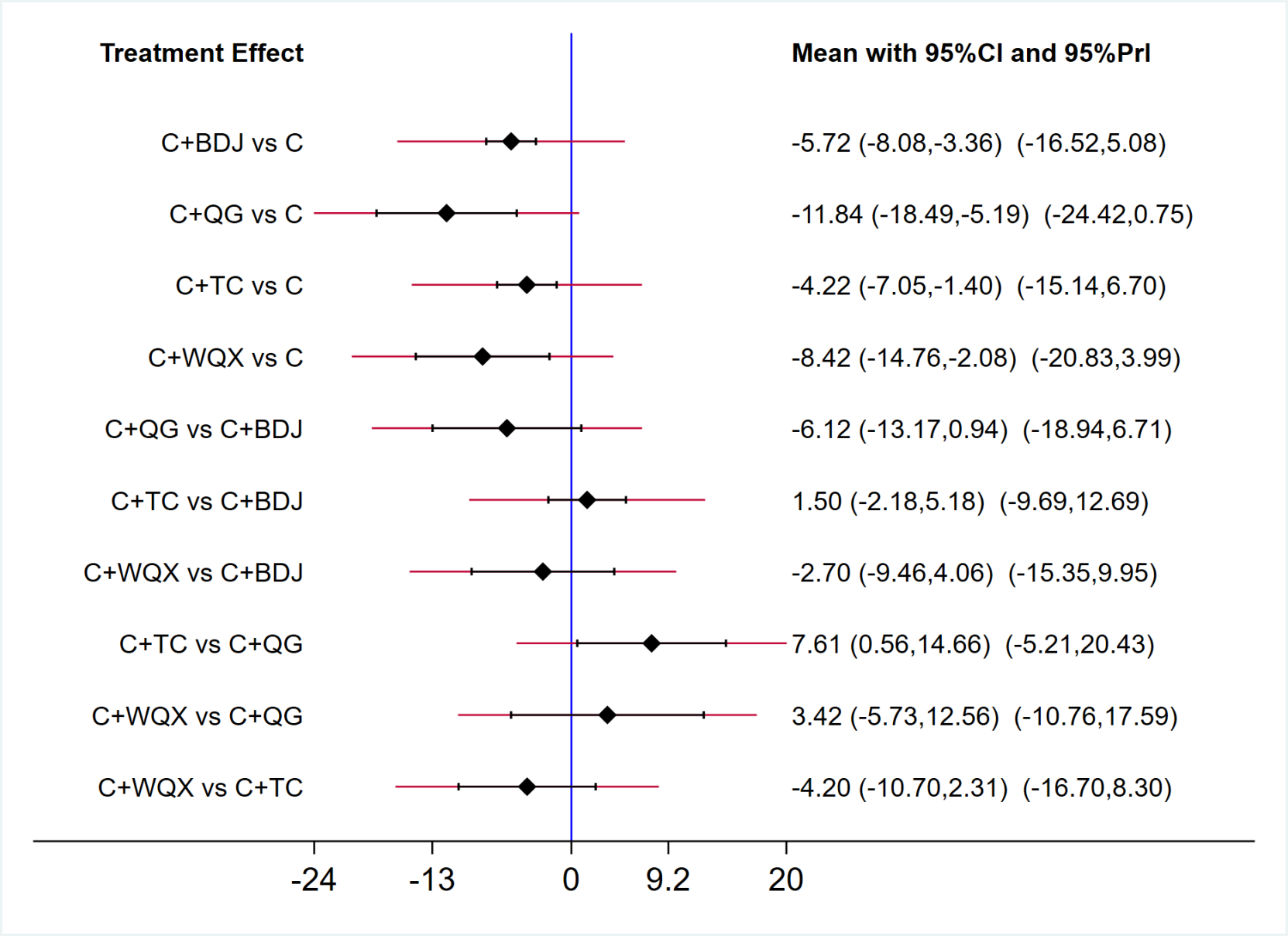


**Figure S2B** DBP.


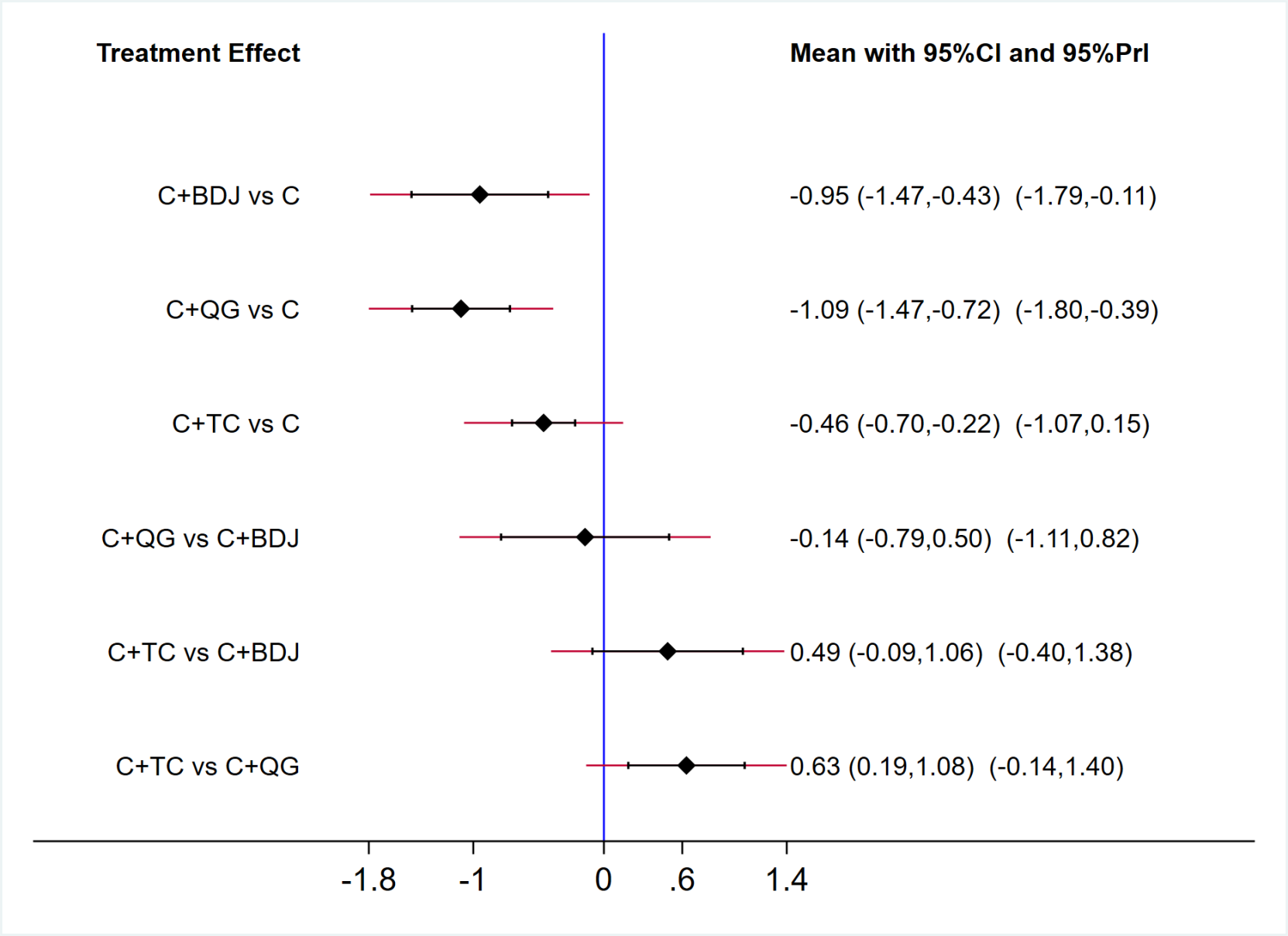


**Figure S2C** TC.


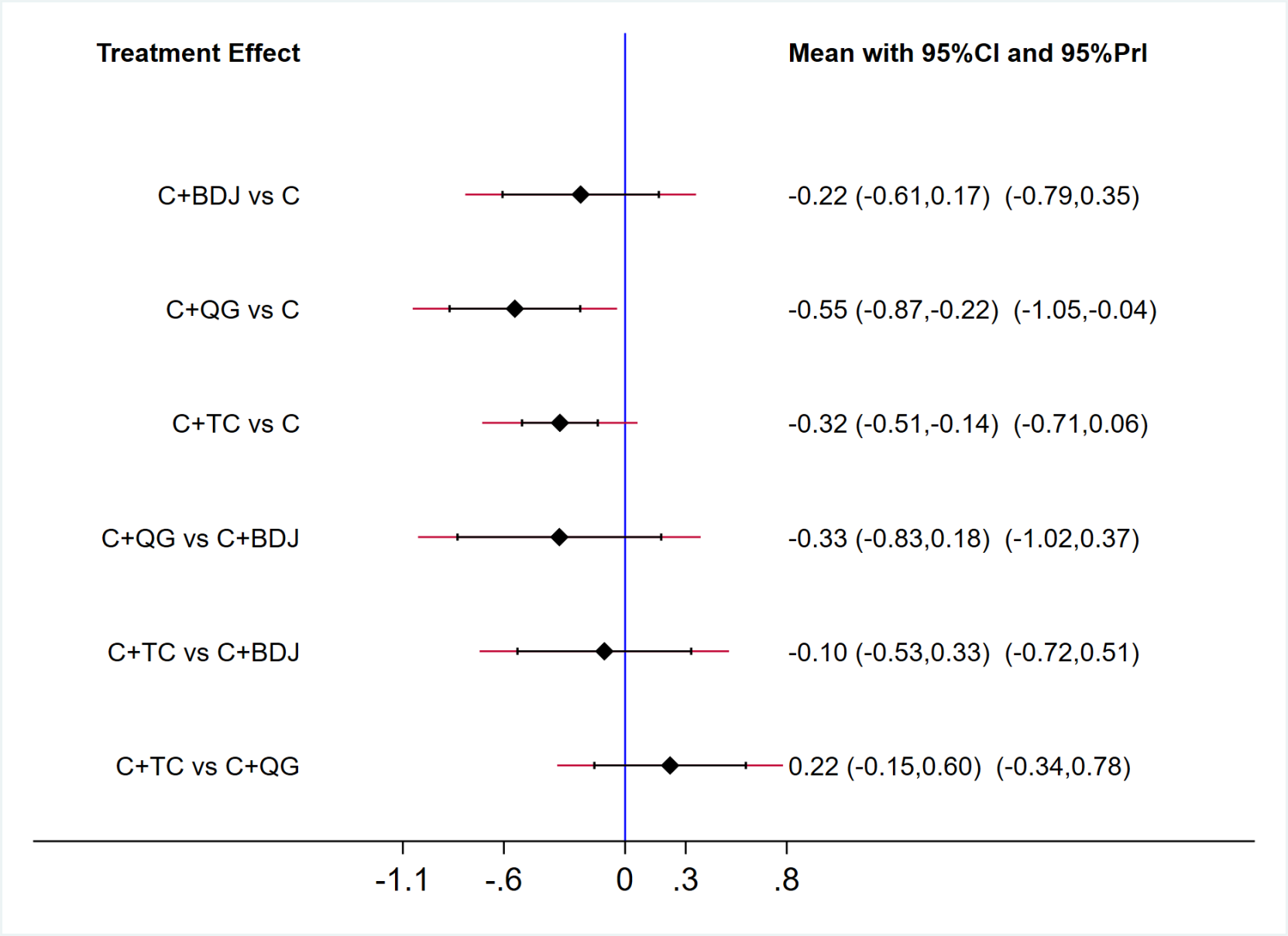


**Figure S2D** TG.


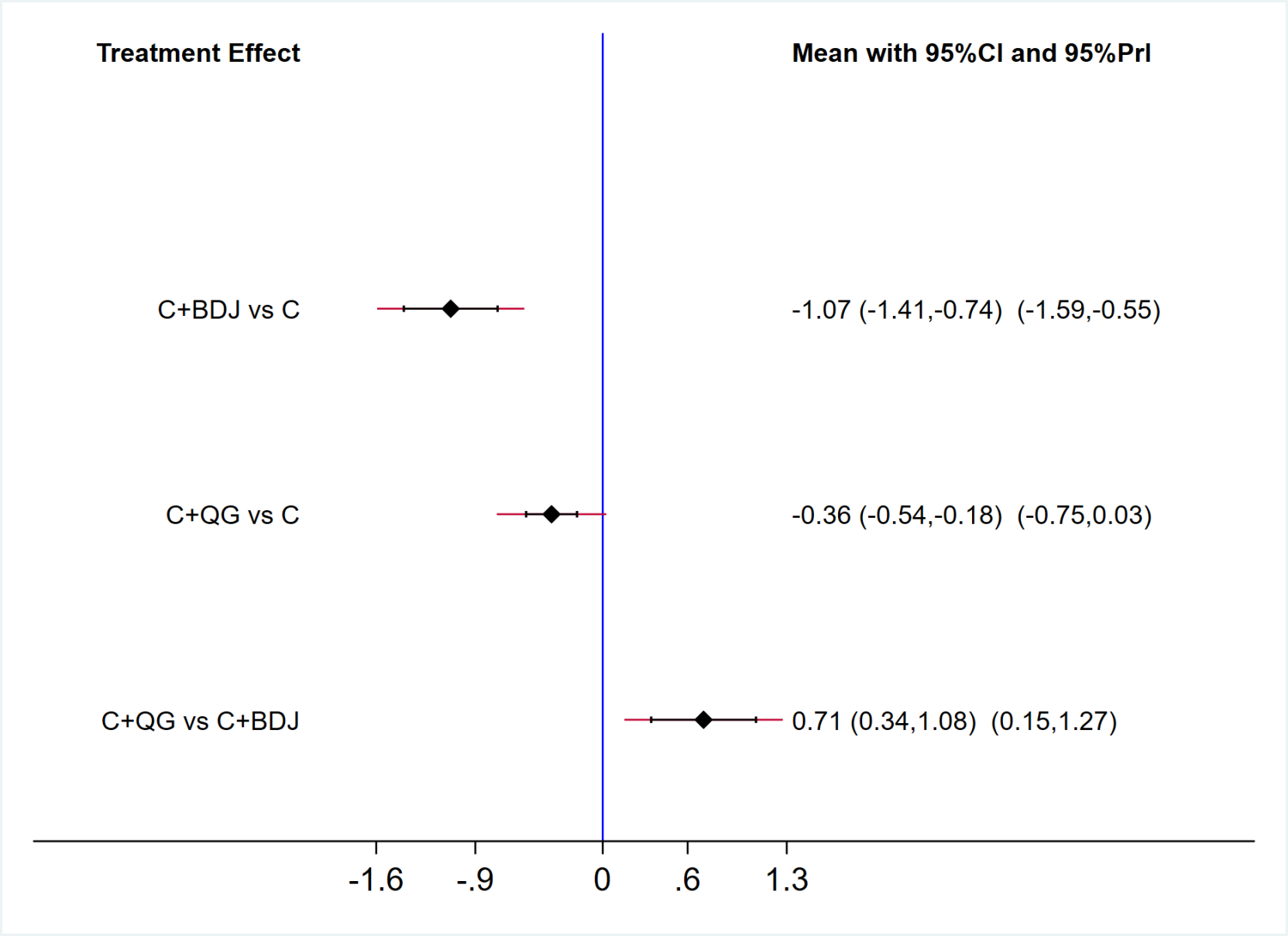


**Figure S2E** LDL-C.


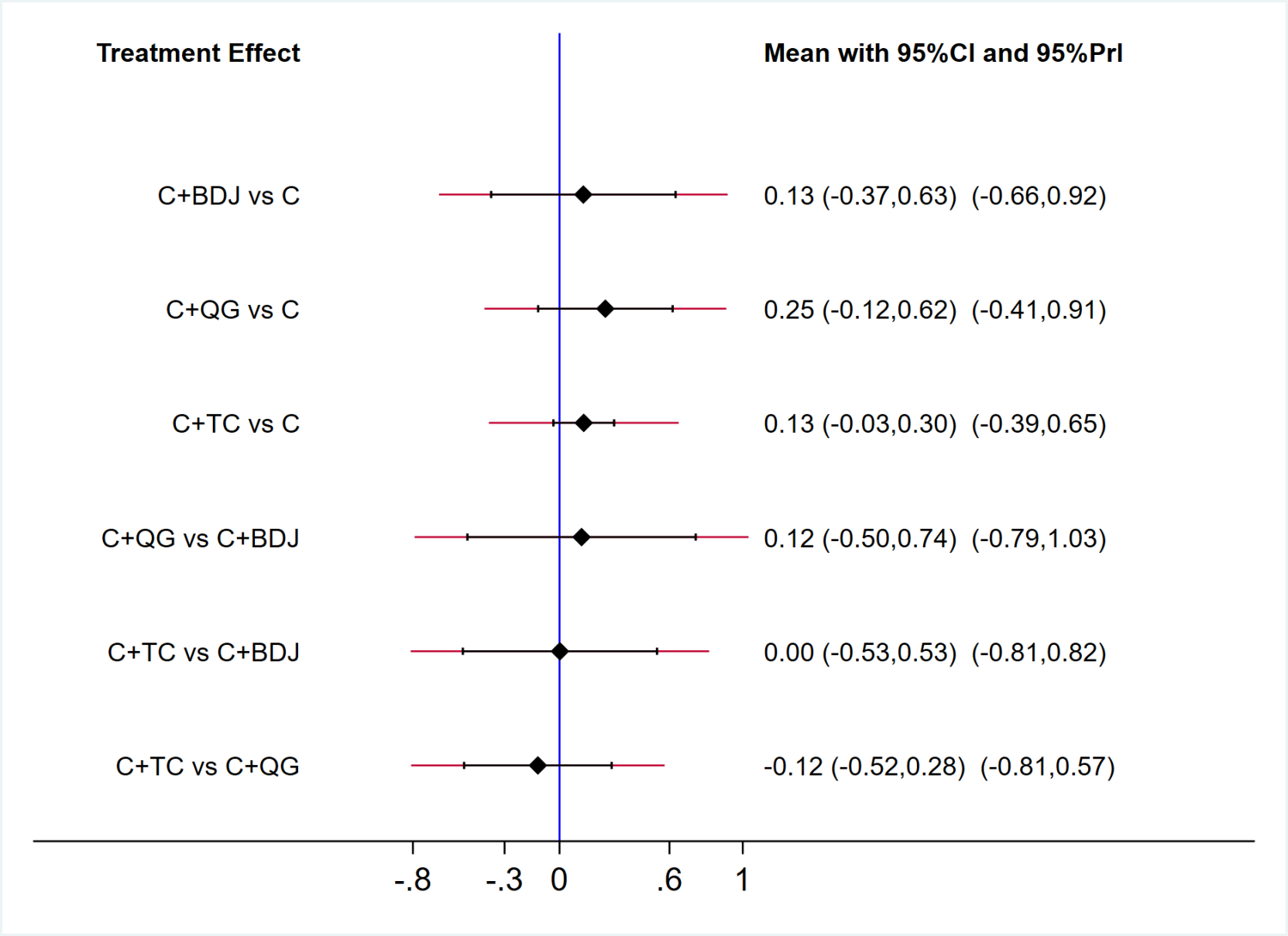


**Figure S2F** HDL-C.

**
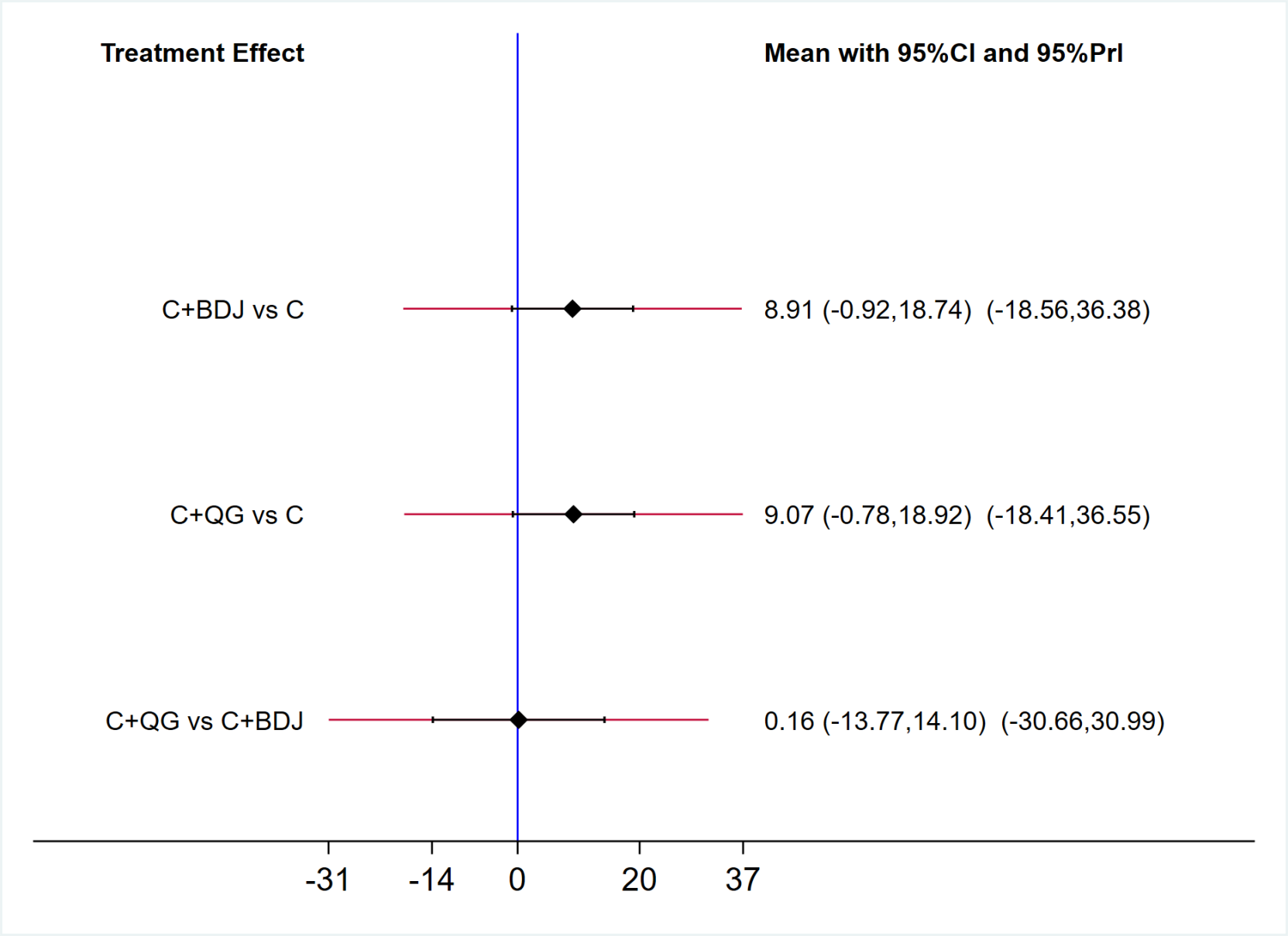
**

**Figure S2G** QOL.

**
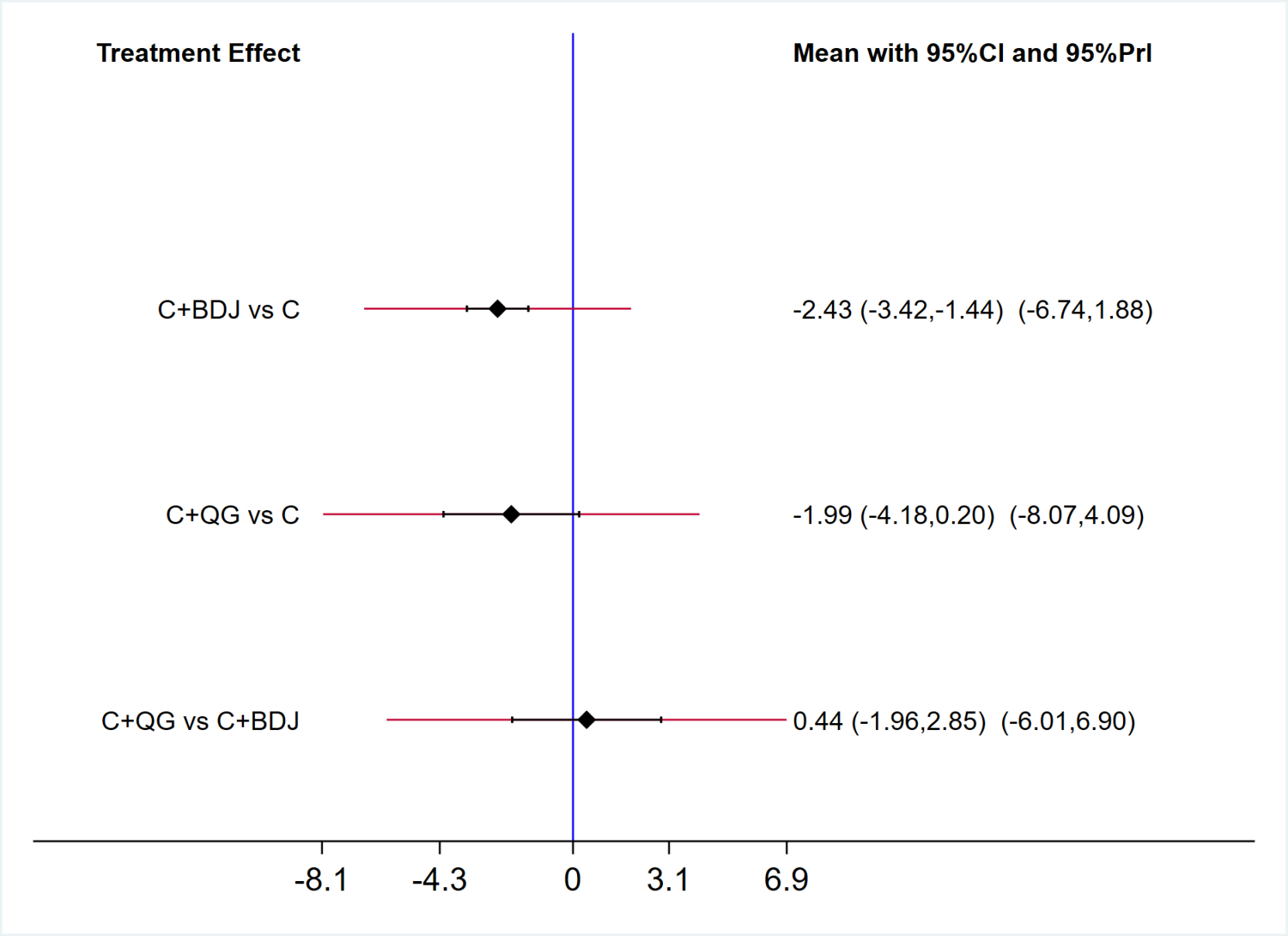
**

**Figure S2H** PSQI.

**Figure S2.** Forest plots of eligible comparisons.

**A.** Systolic Blood Pressure; **B.** Diastolic Blood Pressure; **C.** Total cholesterol; **D.** Triglyceride; **E.** Low-density lipoprotein cholesterol; **F.** High-density lipoproteincholesterol; **G.** Pittsburgh sleep quality index; **H.** Quality of life.

**Note:** A-C, B-C+BDJ, C-C+QG, D-C+TC, E-C+WQX.
