# Supplementary material for: Comparison of the therapeutic effects of traditional Chinese medicine exercise therapies on blood pressure, lipids, and sleep quality among older patients suffering from hypertension: a systematic review and network meta-analysis
Source: Front Cardiovasc Med. 2026 Mar 11;13:1707525. doi: 10.3389/fcvm.2026.1707525 (PMC13013410; doi:10.3389/fcvm.2026.1707525)
Supplement: Supplementary Table S3 — Inconsistency of SBP, DBP, and HR tested by loop-specific heterogeneity estimates, inconsistency model and node splitting analysis. [file Datasheet3.docx]

**Highlights:**

• Different exercises have their own focuses on improving the blood pressure of older patients suffering from hypertension, with the therapeutic effects varying.

• Evidence-based support for personalized exercises is provided.

• Priority is given to WQX and QG for improving blood pressure.
