# Supplementary figures and images for: Comparison of the therapeutic effects of traditional Chinese medicine exercise therapies on blood pressure, lipids, and sleep quality among older patients suffering from hypertension: a systematic review and network meta-analysis
Source: Front Cardiovasc Med. 2026 Mar 11;13:1707525. doi: 10.3389/fcvm.2026.1707525 (PMC13013410; doi:10.3389/fcvm.2026.1707525)

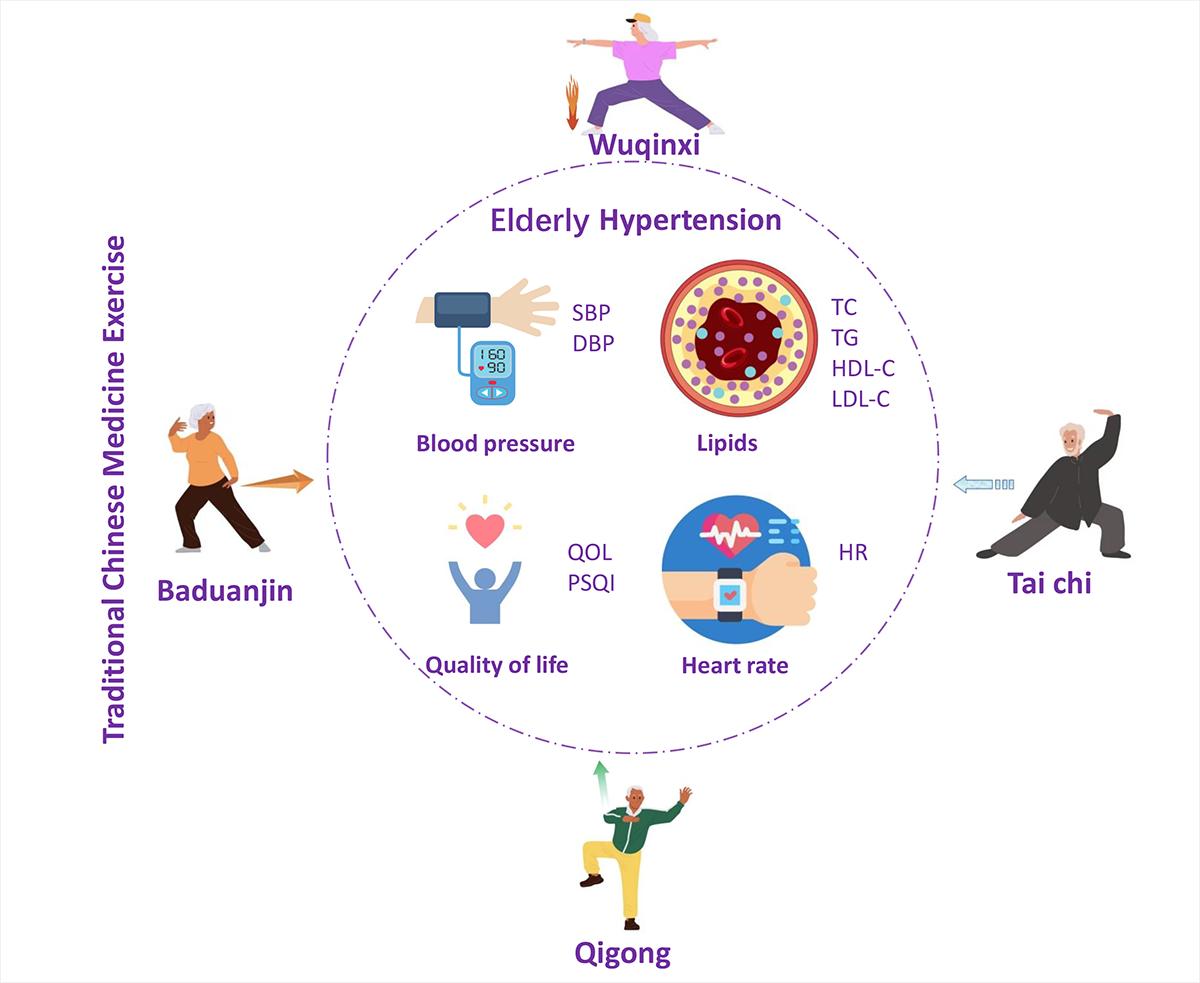

Supplement: Supplementary Figure S1 — Contributions of direct and indirect comparisons to NMA and the number of studies of each direct comparison. (A) Systolic blood pressure; (B) diastolic blood pressure; (C) total cholesterol; (D) triglyceride; (E) low-density lipoprotein cholesterol; (F) high-density lipoproteincholesterol; (G) quality of life; (H) Pittsburgh sleep quality index; and (I) heart rate. [file Image1.tif]
